# Supplementary material for: Cost increase in the electricity supply to achieve carbon neutrality in China
Source: Nat Commun. 2022 Jun 8;13:3172. doi: 10.1038/s41467-022-30747-0 (PMC9177843; doi:10.1038/s41467-022-30747-0)
Supplement: Supplementary file 1 — Supporting Information [file 41467_2022_30747_MOESM1_ESM.pdf]

Cost Increase in the Electricity Supply to Achieve Carbon Neutrality in China (Supplementary Information)

Zhenyu Zhuo[1], Ershun Du[2], Ning Zhang[1,*], Chris P. Nielsen[3], Xi Lu[4], Jinyu Xiao[5], Jiawei Wu[5], and Chongqing Kang[1,*]

[1]Tsinghua University, Department of Electrical Engineering, Beijing, 100084, China
[2]Tsinghua University, Institute of Climate Change and Sustainable Development, Beijing, 100084, China
[3]Harvard University, Harvard-China Project on Energy, Economy and Environment, John A. Paulson School of Engineering and Applied Sciences, Cambridge, Massachusetts 02138, USA.
[4]Tsinghua University, School of Environment, Beijing, 100084, China
[5]Global Energy Interconnection Development and Cooperation Organization, Beijing, 100031, China
[*]Corresponding authors: ningzhang@tsinghua.edu.cn, cqkang@tsinghua.edu.cn

ABSTRACT

This material provides necessary supplemental information for researchers to understand the method and results in the main article. Supplementary Note 1 introduces the formulation of the generation and transmission expansion model (GTEP) in detail. Supplementary Note 2 provides the calculation method of piecewise supply curves and their integration into the GTEP model. The electricity supply costs and marginal carbon prices are presented in Supplementary Note 3 . Supplementary Note 4 presents the sensitivity analysis setting. Other supplementary tables and figures mentioned in the main article are also shown here. Supplementary Note 5 shows details of the VRE potential in China based on our evaluation results. The advantages of our model compared with existing methods are summarized in Supplementary Note 6 .

Contents

Supplementary Note 1 Mathematical Formulation of the Generation and Transmission Expansion Model 3
Supplementary Note 1.1 Assumptions 3
Supplementary Note 1.2 Objective Function 4
Supplementary Note 1.3 Power System Planning Constraints 5
Supplementary Note 1.4 Power System Operation Constraints 7
Supplementary Note 1.5 Solving Method and Code Implementation 11
Supplementary Note 2 Piecewise Supply Curves Calculation 12
Supplementary Note 3 Calculation of Electricity Supply Costs and Carbon Mitigation Costs 14
Supplementary Note 3.1 Electricity Supply Costs 14
Supplementary Note 3.2 Marginal Carbon Prices 14
Supplementary Note 3.3 Average Carbon Mitigation Costs 15
Supplementary Note 4 Sensitivity Analysis Setting 16
Supplementary Note 5 Renewable energy potential and supply curves in China 19
Supplementary Note 6 Comparison with similar existing literature 20
Supplementary Figures 23
Supplementary Tables 36
Supplementary References 50

List of Supplementary Figures

Supplementary Figure 1 **Illustration of piecewise linear fitting for VRE supply curve** 12
Supplementary Figure 2 **Capital cost projection of RE generation technologies for the 30 years.** 17
Supplementary Figure 3 **Average manufacturing capability at each five-year stage.** 17
Supplementary Figure 4 **The electricity load demands under different scenarios** 23

Supplementary Figure 5 The carbon emission limit trajectories under different scenarios 23
Supplementary Figure 6 Technical Procedure for GREAN Platform on VRE Resources Assessment 24
Supplementary Figure 7 Supply curves of wind power in each province 27
Supplementary Figure 8 Supply curves of PV power in each province 30
Supplementary Figure 9 Capacity mix in China under different carbon emission target scenarios 31
Supplementary Figure 10 Transmission network topology under carbon neutrality goals in 2050 32
Supplementary Figure 11 Composition variation of electricity supply cost 33
Supplementary Figure 12 Hourly dispatch schedules in 2020 34
Supplementary Figure 13 Hourly dispatch schedules in 2050 under CN2050 scenario 35

List of Supplementary Tables

Supplementary Table 1 Total electricity energy demands under different scenarios(TWh) 18
Supplementary Table 2 Secure requirement level setting under different scenarios 18
Supplementary Table 3 Total transmission limits of AC and DC candidate lines under different scenarios (GW) 18
Supplementary Table 4 VRE potential and capacity factor distribution in China projected by this study 19
Supplementary Table 5 Comparison with existing articles about the low-carbon transition in China 22
Supplementary Table 6 Abbreviations and regions of provinces in Mainland China 36
Supplementary Table 7 The R-squared value of the transmission line length fitting results for each region . . . 37
Supplementary Table 8 The R-squared value of the transformer capacities fitting results for each region . . . 37
Supplementary Table 9 Projection results of within-province transformer capacities in CN2050 (GW) 38
Supplementary Table 10 Projection results of within-province transformer lines in CN2050 (km) 38
Supplementary Table 11 The data source of Renewable Energy Resources in GREAN platform 39
Supplementary Table 12 The data source of geographic information Resources in GREAN platform 39
Supplementary Table 13 The data source of human activities information in GREAN platform 40
Supplementary Table 14 The parameter of generation technologies 41
Supplementary Table 15 The parameter of energy storage technologies 41
Supplementary Table 16 Variable costs of thermal units in different provinces (CNY/kWh) 42
Supplementary Table 17 The parameter of existing AC transmission lines in 2020 43
Supplementary Table 18 The parameter of candidate AC transmission lines 44
Supplementary Table 19 The parameter of existing DC transmission lines in 2020 45
Supplementary Table 20 The parameter of candidate DC transmission lines 46
Supplementary Table 21 Annual load demands at the provincial level in the NDC/BAU scenario (TWh) 47
Supplementary Table 22 Annual load demands at the provincial level in the GM2.0 scenario (TWh) 48
Supplementary Table 23 Annual load demands at the provincial level in the CN2050 scenario (TWh) 49

Supplementary Note 1 Mathematical Formulation of the Generation and Transmission Expansion Model

This note introduces the mathematical formulation implemented and the corresponding solving algorithm. The multi-stage stochastic optimization model is applied to capture the long-term technology cost changes and the short-term renewable energy intermittent output. The whole model is linearized so that it can be solved in sensible computational time by an off-the-shelter solver. The modelling of components in a power system including various units and transmission networks is described in detail as follows.

On the basis of the roadmap to carbon neutrality, the model simulates the generation and transmission expansion planning (GTEP) from 2020 to 2050 considering the RE potential allocation and current power system configuration in China. The modelling consists of three parts: the objective function, the planning constraints, and the operational constraints. One planning stage covers five years, as does the real scheme in China. The objective function is the total cost of power system investment and operation. The planning constraints represent environment limits and energy policy goals at the macro level, and the operation constraints describe the daily power system dispatch at the micro level. The model optimizes the investment variables and operating variables over the thirty-year planning period globally to avoid the impacts of myopic decision making[1].

Supplementary Note 1.1 Assumptions

To simplify the model and make the optimization problem tractable, the GTEP model is formulated base on the following assumptions:

- The GTEP model is formulated at the provincial level, where each provincial power system is considered as a single bus. The specific topology of the within-province grid is not considered. The within-province grid data are also confidential and not accessible. Regardless of the inaccessibly of data, the optimization problem is too large to solve within a reasonable time considering the precise within-province grid. When studying the nationwide issues, a common and practical approach is to treat the provincial grid as a single node[2–4].

- Generation units of the same type in one province are aggregated as one unit. The distributed generators in the distribution network, such as distributed PV and distributed biomass units, are also aggregated as one unit. It is assumed that they could be dispatched in a centralized manner through aggregators. The energy storage systems (ESSs) and loads are also merged and connected to the provincial buses. The unit commitment is also simplified in the model. The installed capacities and online capacities are both modelled as continuous variables (see the following model formulation for details). We consider eleven kinds of generation units and two kinds of ESSs in the model. The characteristic parameters of these units are presented in Table 14 and Table 15.

- The pipeline model (also called transportation model) is applied to describe the transmission power flow while avoiding the introduction of binary variables[5]. The model assumes that the power of each line can be freely dispatched within the capacity limits. The transmission directions of the DC transmission lines are fixed, and all transmission lines of the same type between any two provincial buses are merged. This method is reasonable when considering a bulk power system with high voltage levels. Moreover, line losses are assumed to be proportional to the online power flow.

- We collected the typical daily load curves of each province for each month in per unit value provided by the National Development and Reform Commission[6]. The per unit curves are scaled up based on annual electrical energy demands and the monthly maximum and minimum load capacity to generate year-around load demand curves with 8760 hourly points. The annual load demands at provincial level for each scenario are provided in Table 21 - Table 23

- The GTEP model is formulated from the perspective of a central planner considering no market behaviour. The investment decisions are made by the central planner pursuing social welfare and carbon emission reduction goals. The total costs involved are then socialized among all users. Since this article focuses on the electricity supply costs brought by carbon neutrality, the profits of various power system entities are not calculated.

- In the GTEP model, the load management strategies are modelled as load shedding. At present, load management strategies (also called demand responses) are only carried out in a few pilots in China. In Tianjin city, the current average price for demand response is approximately 3.3 CNY/kWh. In Jiangsu province, the prices for demand response vary between 1.33 CNY/kWh and 5 CNY/kWh. The cost of load shedding in this paper is set to 3 CNY/kWh according to the Annual Development Report of China's Power Industry 2021[7].

- To represent daily ESS cycles, load demands and RE production correlations, each operation scenario is described by one day, i.e., 24 hours. Twelve typical days are selected from the original full-year hourly dataset according to their numerical characteristics. We conduct the well-known K-medoids algorithm to cluster and select the representative daily scenarios.

- The GTEP model considers only carbon emissions caused by electricity generation and transmission; carbon emissions during the manufacturing process of the units are not taken into account.
- The base year of market prices is set to 2020, which means the prices are normalized based on Chinese Yuan in 2020. In the main article, the exchange rate of CNY to USD is set to 0.144982, which is the average rate in 2020.

Supplementary Note 1.2 Objective Function

The optimization model aims to minimize the sum of investment cost, maintenance cost and operating cost over the planning periods, as follows:

$$\min C = \sum_{y} (1+i)^{-5y} \left(C_y^{\mathrm{INV}} + C_y^{\mathrm{MAT}} + C_y^{\mathrm{OP}}\right) \tag{1}$$

where C_y^{INV}, C_y^{MAT}, and C_y^{OP} denote the investment cost, maintenance cost and operating cost in stage y. The total cost in each stage is converted to the present value at the end of 2020 by the term $(1+i)^{-5y}$, where i is the discount rate.

The investment cost C_y^{INV} is proportional to the capacity increments of units and transmission lines. Specifically, C_y^{INV} consists of the investment cost of generation plants (GP), energy storage systems (ESSs), and transmission lines (TLs), as follows:

$$C_y^{\mathrm{INV,type}} = \sum_{r} \sum_{m \in \Omega^{\mathrm{type}}} \left(\sum_{x=1}^{35-5y} (1+i)^{-x} \right) \frac{i}{1-(1+i)^{-Y_m}} I_{m,y}^{\mathrm{INV,type}} \Delta \mathrm{Ui}_{m,r,y}^{\mathrm{type}}, \quad \mathrm{type} \in \{\mathrm{GP} \backslash \mathrm{VRE}, \mathrm{ESS}, \mathrm{TL}\} \tag{2}$$

where $\Delta \mathrm{Ui}_{m,r,y}^{\mathrm{type}}$ denotes the capacity increment for unit m in bus r at stage y. $I_{m,y}^{\mathrm{INV,type}}$ denotes the investment cost per MW for unit m at stage y. Ω^{type} denotes the set of corresponding devices. Considering the technology developments, the investment costs per MW changes over the planning stages. The overnight investment costs at stage y are converted to the capital costs at the planning period by multiplying $\left(\sum_{x=1}^{35-5y} (1+i)^{-x} \right) \frac{i}{1-(1+i)^{-Y_m}}$. Y_m denotes the lifetime of unit m. For variable renewable energy (VRE, indicating wind and PV in this article) units, the expression is slightly different since the piecewise levelized cost of energy (LCOE) is considered in a piecewise manner. The overnight investment cost for each unit is the sum of the investment costs in all segments, as follows:

$$C_y^{\mathrm{INV,type}} = \sum_{r} \sum_{m \in \Omega^{\mathrm{type}}} \left(\sum_{x=1}^{35-5y} (1+i)^{-x} \right) \frac{i}{1-(1+i)^{-Y_m}} \left(\sum_{s} I_{m,r,y,s}^{\mathrm{INV,type}} \Delta \mathrm{Ui}_{m,r,y,s}^{\mathrm{type}} \right), \quad \mathrm{type} \in \{\mathrm{VRE}\} \tag{3}$$

where $\Delta \mathrm{Ui}_{m,r,y,s}^{\mathrm{type}}$ denotes the capacity increment of segment s for unit m in bus r at stage y. $I_{m,y,s}^{\mathrm{INV,type}}$ is the equivalent overnight investment cost per MW in segment s, which is obtained by the piecewise linear method described in Supplementary Note 2 .

The maintenance cost C_y^{MAT} is proportional to the existing capacity of units and transmission lines in each stage. Similarly, C_y^{MAT} consists of the three parts from GP, ESS and TL, as follows:

$$C_y^{\mathrm{MAT,type}} = \sum_{r} \sum_{m \in \Omega^{\mathrm{type}}} \left(\sum_{x=1}^{5} (1+i)^{-x} \right) I_{m,y}^{\mathrm{MAT,type}} U_{m,r,y}^{\mathrm{type}}, \quad \mathrm{type} \in \{\mathrm{GP}, \mathrm{ESS}, \mathrm{TL}\} \tag{4}$$

The operating costs include the penalty costs of load shedding in each region, the power generation costs, and the start-up costs for each unit. The CO_2 capture costs are also included when carbon capture and storage (CCS) units are involved. Twelve representative operating days are selected from the year-round dataset via the scenario reduction method (K-medoids method) to make the optimization problem tractable. The operating costs are calculated based on the system conditions over the representative day set. The expressions of each part are as follows:

$$C_y^{\text{OP,GP}} = \sum_{m\in\Omega^{\text{GP}}} \sum_r \sum_d \varphi_d \sum_t c_m P_{m,r,y,d,t} \tag{5}$$

$$C_y^{\text{OP,ON}} = \sum_{m\in\Omega^{\text{GP}}} \sum_r \sum_d \varphi_d \sum_t c_m^{\text{on}} \Delta \text{Ud}_{m,r,y,d,t}^{\text{on}} \tag{6}$$

$$C_y^{\text{OP,Lshed}} = \sum_r \sum_d \varphi_d \sum_t c_r^{\text{Lshed}} P_{r,y,d,t}^{\text{Lshed}} \tag{7}$$

$$C_y^{\text{OP,CO}_2} = \sum_{m\in\Omega^{\text{CCS}}} \sum_r \sum_d \varphi_d \sum_t c_r^{\text{cap}} E_{r,y,d,t}^{\text{cap}} \tag{8}$$

$$C_y^{\text{OP}} = C_y^{\text{OP,GP}} + C_y^{\text{OP,ON}} + C_y^{\text{OP,Lshed}} + C_y^{\text{OP,CO}_2} \tag{9}$$

where φ_d denotes the number of duration days of the representative day d within one five-year stage and $E_{r,y,d,t}^{\text{cap}}$ is the weight of CO_2 captured by CCS units. The carbon capture costs c_r^{cap} for Coal-CCS, Gas-CCS, and Bio-CCS are set to 390.8 CNY/tonne, 305.4 CNY/tonne, and 305.4 CNY/tonne respectively[8].

Supplementary Note 1.3 Power System Planning Constraints

The planning constraints, such as the upper limit of renewable energy units and expansion speed constraints, represent the limits and variable relationships over planning stages. Environmental policy boundaries, such as CO_2 emission reduction and renewable penetration goals, are also included. This section introduces these constraints in detail.

- Total Developable Renewable Capacity Constraints

For renewable energy units such as hydro units(HU), VRE units, and biomass power units(BU), we consider the available resources in different provinces and regions. Hence, there is a constraint on the maximum technological potential obtained from the GREAN database. Nuclear unit (NU) capacities are also limited due to policy requirements and unaffordable accident impacts. This constraint is formulated as:

$$U_{m,r,y}^{\text{GP}} \le \overline{U}_{m,r}^{\text{GP}}, \forall m \in \left\{\Omega^{\text{HU}}, \Omega^{\text{VRE}}, \Omega^{\text{BU}}, \Omega^{\text{NU}}\right\}, y \tag{10}$$

where $\overline{U}_{m,r}^{\text{GP}}$ is the upper limit of generation plants for technology m in region r.

- Continuity Equation Constraints of Installed Capacity

For different units, the installed capacity at a certain planning stage depends on the installed capacity at the previous stage, the expansion in capacity and the retired capacity at the current stage. The capacity increments in each region are constrained due to the limits on construction ability and environmental issues. The following continuity constraints are met:

$$U_{m,r,y}^{\text{type}} = U_{m,r,y-1}^{\text{type}} + \Delta\text{Ui}_{m,r,y}^{\text{type}} - \Delta\text{Ud}_{m,r,y}^{\text{type}}, \forall m,r,y, \text{type} \in \{\text{GP}, \text{ESS}, \text{TL}\} \tag{11}$$

$$0 \le \Delta\text{Ui}_{m,r,y}^{\text{type}} \le \Delta\overline{\text{Ui}}_{m,r,y}^{\text{type}}, \forall m,r,y, \text{type} \in \{\text{GP}, \text{ESS}, \text{TL}\} \tag{12}$$

$$0 \le \Delta\text{Ud}_{m,r,y}^{\text{type}} \le \Delta\overline{\text{Ud}}_{m,r,y}^{\text{type}}, \forall m,r,y, \text{type} \in \{\text{GP}, \text{ESS}, \text{TL}\} \tag{13}$$

$$U_{m,r,0}^{\text{type}} \le U_{m,r,y}^{\text{type}}, \forall m,r,y, \text{type} \in \{\text{TL}\} \tag{14}$$

where $U_{m,r,0}^{\text{type}}$ is the capacities of existing lines and (14) means the transmission lines are not allowed to be retired. $\Delta\text{Ud}_{m,r,y}^{\text{type}}$ denotes the capacity decrement for unit m in bus r at stage y. The expansion and retiring speeds are limited by (12) and (13).

- Piecewise Development Constraints for Wind and PV Units

As discussed previous, the development cost distribution curve of wind and PV is integrated into the optimization model in a piecewise manner. The VRE potential in each province is divided into several segments according to the development costs. The installed capacity in each segment must satisfy the following constraints

$$U_{m,r,y,s}^{\text{type}} = U_{m,r,y-1,s}^{\text{type}} + \Delta\text{Ui}_{m,r,y,s}^{\text{type}} - \Delta\text{Ud}_{m,r,y,s}^{\text{type}}, \forall m,r,y,s, \text{type} \in \{\text{VRE}\} \tag{15}$$

$$0 \le U_{m,r,y,s}^{\text{type}} \le \overline{U}_s^{\text{type}}, \forall m,r,y,s, \text{type} \in \{\text{VRE}\} \tag{16}$$

$$U_{m,r,y}^{\text{type}} = \sum_s U_{m,r,y,s}^{\text{type}}, \forall m,r,y,s, \text{type} \in \{\text{VRE}\} \tag{17}$$

where $U^{\text{type}}_{m,r,y,s}$ denotes the installed capacity of segment s for unit m in bus r at stage y. $\Delta \text{Ud}^{\text{type}}_{m,r,y,s}$ denotes the capacity decrements of segment s for unit m in bus r at stage y. $\overline{U}^{\text{type}}_{s}$ is the upper limit of the capacity in segment s, which is obtained by the piecewise linear method described in Supplementary Note 2 .

- Natural Gas Resource Constraints

The supply of natural gas resources in China is tight and heavily dependent on imports. Hence, generation unit (GU) expansion planning must consider the natural gas resource constraints faced by each province. The amount of natural gas used for power generation cannot exceed the available natural gas power generation resources in the region. The gas networks are also modelled:

$$\sum_{m\in\Omega^{\text{GU}}} \eta_m \sum_{d} \varphi_d \sum_{t} P_{m,r,y,d,t} \leq \Gamma_{r,y} + \Gamma^{\text{import}}_{r,y} - \Gamma^{\text{export}}_{r,y}, \forall r, y \tag{18}$$

$$0 \leq \Gamma^{\text{import}}_{r,y} \leq \overline{\Gamma}^{\text{import}}_{r,y}, \forall r, y \tag{19}$$

$$0 \leq \Gamma^{\text{export}}_{r,y} \leq \overline{\Gamma}^{\text{export}}_{r,y}, \forall r, y \tag{20}$$

where η_m denotes the gas consumption per MWh for the technology m. $\Gamma_{r,y}$ denotes the natural gas supply for power generation in the region r including the local production and imports from foreign countries. The gas import $\Gamma^{\text{import}}_{r,y}$ and export $\Gamma^{\text{export}}_{r,y}$ are limited by the capacity of natural gas pipeline. In this study, $\Gamma^{\text{import}}_{r,y}$ and $\Gamma^{\text{export}}_{r,y}$ are set to zero because most of the annual natural gas supply for power generation in each province of China is scheduled and fixed.

- Water Constraints

For each provincial region, the total power generation water consumption at each planning stage cannot exceed the given power generation water consumption limit, that is:

$$\sum_{m} \sum_{d} \varphi_d \sum w_m P_{m,r,y,d,t} \leq \overline{W}_{r,y}, \forall r, y \tag{21}$$

where w_m denotes the water consumption per MWh for technology m.

- Generator Capacity Factor Constraint

During unit operation, because of shutdowns caused by maintenance or limited generation resources, it is impossible to guarantee that a unit will always operate at the rated maximum output throughout the year. For example, the annual power generation of hydropower is limited by the water storage of reservoirs. Hence, there is a constraint on the annual maximum capacity factor $\text{cf}^{\max}_{m}$. Furthermore, due to policy requirements or the necessary conditions of plant operation, the generation capacity factor of the unit should not be less than the annual minimum capacity factor $\text{cf}^{\min}_{m}$. Therefore, all the units must satisfy the following constraints:

$$U^{\text{type}}_{m,r,y} \text{cf}^{\min}_{m} \leq \sum_{d} \varphi_d \sum_{t} P_{m,r,y,d,t}/8760 \leq U^{\text{type}}_{m,r,y} \text{cf}^{\max}_{m}, \forall m, r, y, \text{type} \in \text{GP} \tag{22}$$

- Carbon Emission Reduction Goals

The total CO_2 emitted by the electrical sector is supposed to meet the given carbon emission reduction goals, that is:

$$\sum_{r} \sum_{d} \varphi_d \left(\sum_{m\in\Omega^{\text{TU}}/\Omega^{\text{CCS}}} \sum_{t} e_m P_{m,r,y,d,t} + \sum_{m\in\Omega^{\text{CCS}}} \sum_{t} E^{\text{net,CCS}}_{m,r,y,d,t} \right) \leq E_y, \forall y \tag{23}$$

- Power Reserve Requirements

The unit capacity in each region is supposed to be larger than the local peak load to guarantee security under unexpected accidents. The proportion of the excess part is called the reserve rate, which needs to meet the requirements in each provincial power system. The expressions is as follows:

$$\sum_{m\in\left\{\Omega_r^{\mathrm{GP}},\Omega_r^{\mathrm{ESS}}\right\}} r_m U_{m,r,y} + \sum_{m\in\Omega_r^{\mathrm{DC,to}}} U_{m,y}^{\mathrm{DC}} \geq (1+\mathrm{rs}_r)\left(\max_{d,t} L_{r,y,d,t} + \sum_{m\in\Omega_r^{\mathrm{DC,from}}} U_{m,y}^{\mathrm{DC}}\right), \forall r,y \tag{24}$$

where rs_r is the required reserve rate in region r (around 13%-15%) and r_m is the credit capacity rate of generation technology m. Since the outputs of renewable energy units, such as wind and PV, are intermittent and not dispatchable, their credit capacity rates are lower than those of conventional units. The item on the left of the inequality sign represents the power reserve provided by the local generators and the transmission grid, and the item on the right represents the local reserve demand in the region r (Note that a region corresponds to a province here). $r_m U_{m,r,y}$ denotes the power reserve capacities provided by the local generation plants of technology m in region r at stage y. $U_{m,y}^{\mathrm{DC}}$ denotes the the power reserve capacities by the DC transmission lines. $\Omega_r^{\mathrm{DC,to}}$ denotes the subset of the whole DC transmission lines whose receiving end is region r. The local reserve demand is proportional to the peak load $\max_{d,t} L_{r,y,d,t}$ and the reserve of other regions brought by the DC transmission line. $\Omega_r^{\mathrm{DC,to}}$ denotes the subset of the whole DC transmission lines whose sending end is region r. rs_r is the required reserve rate in region r. In power system operation, the sending end deploys dedicated power plants for the DC lines and the receiving end regards the DC lines as dispatchable units. Hence, DC transmission lines can transfer the power reserve capacities between the provinces in our model. For AC transmission lines that connect two provinces, their power flow is coordinately controlled by the dispatching centres on both sides using the Area Control Error (ACE) criteria which are generated based on the supply-demand imbalance of each province. Therefore, there is no a determined receiving end or a sending end of the reserve capacity for AC transmission lines. Consequently, AC transmission lines are not regarded to transfer reserves between provinces.

Supplementary Note 1.4 Power System Operation Constraints

The operation constraints model the generation characteristics of units and power balancing. These constraints simulate the daily dispatch in the selected representative days whose modelling is performed at the hour level.

- Regional Power Balancing

At each time period in a representative day, each provincial system must meet the power load balance constraints, as follows:

$$\begin{aligned}&\sum_{m\in\Omega^{\mathrm{GP}}\setminus\Omega^{\mathrm{CCS}}} P_{m,r,y,d,t} + \sum_{m\in\Omega^{\mathrm{CCS}}} P_{m,r,y,d,t}^{\mathrm{net,CCS}} + \sum_{m\in\Omega^{\mathrm{ESS}}} (P_{m,r,y,d,t}^{\mathrm{dis}} - P_{m,r,y,d,t}^{\mathrm{ch}}) - \sum_{m\in\Omega_r^{\mathrm{AC,from}}} f_{m,y,d,t}^{\mathrm{AC,from}} - \sum_{m\in\Omega_r^{\mathrm{DC,from}}} f_{m,y,d,t}^{\mathrm{DC,from}}\\&+\sum_{m\in\Omega_r^{\mathrm{AC,to}}} f_{m,y,d,t}^{\mathrm{AC,to}} + \sum_{m\in\Omega_r^{\mathrm{DC,to}}} f_{m,y,d,t}^{\mathrm{DC,to}} = (D_{r,y,d,t} - P_{r,y,d,t}^{\mathrm{Lshed}})/(1-l_r^{\mathrm{local}}), \forall r,y,d,t\end{aligned} \tag{25}$$

where l_r^{local} denotes the losses of the within-province grid in region r. $\Omega_r^{\mathrm{AC,from}}$ and $\Omega_r^{\mathrm{DC,from}}$ are the sets of AC and DC lines, respectively, where region r is the from bus. $\Omega_r^{\mathrm{AC,to}}$ and $\Omega_r^{\mathrm{DC,to}}$ are the sets of AC and DC lines, respectively, where region r is the to bus. Moreover, the capacity of load shedding cannot exceed the load demand.

$$0 \leq P_{r,y,d,t}^{\mathrm{Lshed}} \leq D_{r,y,d,t}, \forall r,y,d,t \tag{26}$$

- Transmission Line Model

To handle the complex constraints introduced by the power flow equation, this paper applies a pipeline model (also called transportation model) to avoid the introduction of binary variables[5]. The pipeline model assumes that the power of each line can be freely dispatched within the capacity limits. This method is demonstrated to be reasonable for the power exchange between provinces when considering the national transmission power system planning with high voltage levels. Currently, many planning studies for national or regional power systems have adopted the pipeline model[3,9–13]. In our model, each bus corresponds to an aggregation of a provincial power grid, not a real bus in the power system as stated in Supplementary Note 1.1. Thus, the free control of power flow on the AC transmission lines can be achieved by the line switch operation or the

coordinated dispatch of reactive and active power within the province grid. For DC transmission lines, free control is their inherent advantage thanks to the power-electronic control technologies. Hence, the pipeline model is reasonable for our case. The modelling of power losses is also smoother when applying the pipeline model. Line losses are assumed to be proportional to the online power flow. The expressions for AC transmission lines are as follows:

$$f_{m,y,d,t}^{\text{AC,from}} = f_{m,y,d,t}^{\text{AC,forward}} - (1 - l_m^{\text{AC}}) f_{m,y,d,t}^{\text{AC,back}}, \forall m \in \left\{\Omega^{AC}\right\}, y, d, t \tag{27}$$

$$f_{m,y,d,t}^{\text{AC,to}} = (1 - l_m^{\text{AC}}) f_{m,y,d,t}^{\text{AC,forward}} - f_{m,y,d,t}^{\text{AC,back}}, \forall m \in \left\{\Omega^{AC}\right\}, y, d, t \tag{28}$$

$$0 \leq f_{m,y,d,t}^{\text{AC,forward}}, f_{m,y,d,t}^{\text{AC,back}} \leq U_{m,y}^{\text{AC}}, \forall m \in \left\{\Omega^{AC}\right\}, y, d, t \tag{29}$$

where $f_{m,y,d,t}^{\text{AC,forward}}$ and $f_{m,y,d,t}^{\text{AC,back}}$ are the two auxiliary variables to model the AC line losses and l_m^{AC} denotes the line loss rate, which is set as the empirical value. Eq. (29) requires that power flow does not exceed the line capacity. The modelling equation of DC lines is similar. The only difference is that only one-way power flow is allowed on DC lines since most cross-region HVDC lines are LCC type, which can only operate in unidirectional mode. The expressions are as follows:

$$f_{m,y,d,t}^{\text{DC,from}} = f_{m,y,d,t}^{\text{DC,forward}}, \forall m \in \left\{\Omega^{DC}\right\}, y, d, t \tag{30}$$

$$f_{m,y,d,t}^{\text{DC,to}} = (1 - l_m^{\text{DC}}) f_{m,y,d,t}^{\text{DC,forward}}, \forall m \in \left\{\Omega^{DC}\right\}, y, d, t \tag{31}$$

$$0 \leq f_{m,y,d,t}^{\text{DC,forward}} \leq U_{m,y}^{\text{DC}}, \forall m, y, d, t \tag{32}$$

- Thermal and Nuclear Power Generation Constraints

Coal power, gas power, and biomass energy are all considered thermal units(TUs) in this paper. The outputs of thermal units and nuclear units (NUs) are clipped by the online capacity and the technical minimum output as presented in (33). The online capacity cannot exceed the installed capacity.

$$\kappa_m U_{m,r,y,d,t}^{\text{on}} \leq P_{m,r,y,d,t} \leq U_{m,r,y,d,t}^{\text{on}}, \forall m \in \{\Omega^{\text{TU}}, \Omega^{\text{NU}}\}, r, y, d, t \tag{33}$$

$$0 \leq U_{m,r,y,d,t}^{\text{on}} \leq U_{m,r,y}, \forall m \in \{\Omega^{\text{TU}}, \Omega^{\text{NU}}\}, r, y, d, t \tag{34}$$

$$U_{m,r,y,d,t}^{\text{on}} - U_{m,r,y,d,t-1}^{\text{on}} = \Delta \text{Ui}_{m,r,y,d,t}^{\text{on}} - \Delta \text{Ud}_{m,r,y,d,t}^{\text{on}}, \forall m \in \{\Omega^{\text{TU}}, \Omega^{\text{NU}}\}, r, y, d, t \tag{35}$$

$$0 \leq \Delta \text{Ud}_{m,r,y,d,t}^{\text{on}}, \Delta \text{Ui}_{m,r,y,d,t}^{\text{on}}, \forall m \in \{\Omega^{\text{TU}}, \Omega^{\text{NU}}\}, r, y, d, t \tag{36}$$

where κ_m is the minimum output rate of technology m. (35) and (36) represents the start-up capacities $\Delta \text{Ui}_{m,r,y,d,t}^{\text{on}}$ and shut-down capacities $\Delta \text{Ud}_{m,r,y,d,t}^{\text{on}}$ at time period t. The start-up capacities are the capacity of units newly turned on at time period t, and shut-down capacities are the capacity of units turned off at time period t. Since this model simulates only daily operation, the initial online capacity $U_{m,r,y,d,0}^{\text{on}}$ is given as follows:

$$U_{m,r,y,d,0}^{\text{on}} = \text{cf}_m U_{m,r,y}, \forall m \in \Omega^{\text{TU}}, r, y, d, t \tag{37}$$

where cf_m is the capacity factors of thermal generators of technology m. Additionally, the output of the nuclear power plant is not allowed to change within one representative day:

$$P_{m,r,y,d,t} = P_{m,r,y,d,t-1}, \forall m \in \Omega^{\text{NU}}, r, y, d, t \tag{38}$$

- The model of CCS units

CCS units can capture CO2 through carbon capture devices installed in thermal plants. Hence, they produce less carbon than do conventional thermal power plants. However, carbon capture devices are expensive, and the capture process consumes considerable power. Therefore, the net output of CCS plant $P_{m,r,y,d,t}^{\text{net,CCS}}$ is less than the actual power generated. Thus, the CCS unit can adjust its CO2 capture strength to change the emission net electricity output and carbon emission. In addition to the conventional operation constraints of thermal power units described in (33)-(37), the CCS unit should also satisfy the following constraints:

$$P_{m,r,y,d,t}-P^{\text{net,CCS}}_{m,r,y,d,t}=\lambda^{\text{CCS}}_{m}E^{\text{cap}}_{m,r,y,d,t}+P^{\text{BA}}_{m,r,y},\forall m\in\Omega^{\text{CCS}},r,y,d,t \tag{39}$$

$$0\leq E^{\text{cap}}_{m,r,y,d,t}\leq \rho_m e_m P_{m,r,y,d,t},\forall m\in\Omega^{\text{CCS}},r,y,d,t \tag{40}$$

$$E^{\text{net,CCS}}_{m,r,y,d,t}=e_m P_{m,r,y,d,t}-E^{\text{cap}}_{m,r,y,d,t}\forall m\in\Omega^{\text{CCS}},r,y,d,t \tag{41}$$

where ρ_m denotes the maximum capture rate, with a typical value between 80 % and 95 %: it is taken as 90 % in this paper. λ^{CCS}_{m} denotes the required power for CO_2 capture and storage: the typical value is 0.296 MWh per tonne[14]. $P^{\text{BA}}_{m,r,y}$ is the basic energy consumption of the CCS unit, which is independent of the operating state and is approximately 0.5% of the CCS unit capacity.

- Wind and PV Power Generation Constraints

For intermittent generation units such as wind power and PV, the actual output during operation cannot exceed the maximum generation output, that is:

$$0\leq P_{m,r,y,d,t}\leq \omega^{\text{VRE}}_{m,r,y,d,t}U_{m,r,y},\forall m\in\Omega^{\text{VRE}},r,y,d,t \tag{42}$$

where $\omega^{\text{IRE}}_{m,r,y,d,t}$ denotes the maximum generation output at time period t.

- Energy Storage System Model

Pumped hydro storage and battery storage are considered in the model. The following constraints need to be met during operation:

$$0\leq P^{\text{ch}}_{m,r,y,d,t},P^{\text{dis}}_{m,r,y,d,t}\leq U^{\text{ESS}}_{m,r,y},\forall m,r,y,d,t \tag{43}$$

$$S^{\text{ESS}}_{m,r,y,d,t}=S^{\text{ESS}}_{m,r,y,d,t-1}+\eta^{\text{ESS}}_{m}P^{\text{ch}}_{m,r,y,d,t}-P^{\text{dis}}_{m,r,y,d,t}/\eta^{\text{ESS}}_{m},\forall m,r,y,d,t \tag{44}$$

$$S^{\text{ESS}}_{m,r,y,d,t=0}=S^{\text{ESS}}_{m,r,y,d,t=T},\forall m,r,y,d \tag{45}$$

$$0\leq S^{\text{ESS}}_{m,r,y,d,t}\leq T^{\text{ESS}}_{m}U^{\text{ESS}}_{m,r,y},\forall m,r,y,d,t \tag{46}$$

where η^{ESS}_{m} denotes the efficiency of storage discharging and discharging and T^{ESS}_{m} is the maximum duration hours. The time sequential relationship between energy and power of ESS is presented in (44). (45) requires the energy in the storage system to be unchanged after the intraday dispatch. Moreover, the discharging and charging power cannot exceed the power component capacity, and the stored energy cannot exceed the energy component capacity.

- Concentrated Solar Power Model

Concentrated solar power (CSP) plants consist of three parts: the solar heat collection device, the heat storage, and the power generation turbine. The energy transfer between each component is realized through the heat-transfer medium. The operation is as follows:

$$Cap^{\text{SF}}_{m,r,y}=\delta^{\text{SF}}_{m}U^{\text{P}}_{m,r,y}/\eta^{\text{PB}}_{m},\forall m\in\Omega^{\text{CSP}},r,y \tag{47}$$

$$Cap^{\text{TES}}_{m,r,y}=T^{\text{TES}}_{m}U^{\text{P}}_{m,r,y}/\eta^{\text{PB}}_{m},\forall m\in\Omega^{\text{CSP}},r,y \tag{48}$$

$$\left(S^{\text{TES}}_{m,r,y,d,t}-S^{\text{TES}}_{m,r,y,d,t-1}\right)+P_{m,r,y,d,t}/\eta^{\text{PB}}_{m}\leq \omega^{\text{SF}}_{m,r,y,d,t}Cap^{\text{SF}}_{m,r,y},\forall m\in\Omega^{\text{CSP}},r,y,d,t \tag{49}$$

$$S^{\text{TES}}_{m,r,y,d,t=0}=S^{\text{TES}}_{m,r,y,d,t=T},\forall m\in\Omega^{\text{CSP}},r,y,d \tag{50}$$

$$0\leq S^{\text{TES}}_{m,r,y,d,t}\leq Cap^{\text{TES}}_{m,r,y},\forall m\in\Omega^{\text{CSP}},r,y,d,t \tag{51}$$

$$0\leq P_{m,r,y,d,t}\leq U_{m,r,y},\forall m\in\Omega^{\text{CSP}}r,y,d,t \tag{52}$$

where η^{PB}_{m} denotes the thermoelectric conversion efficiency and $\omega^{\text{SF}}_{m,r,y,d,t}$ denotes the capacity factor at time period t. (47) and (48) calculate the maximum capacity of the heat collection devices and heat storage, respectively. (49) - (52) represent the model of the heat storage components of CSPs, which are similar to those in the ESS station model. The only difference is in (49), where the charging power is limited by the maximum capacity of the heat collection devices at time period t.

- Hydro Plant Operation Constraints

The actual output of hydropower units should not exceed the total installed capacity during operation. Notably, the annual energy generation constraint of hydropower is reflected in (22).

$$0 \le P_{m,r,y,d,t} \le U_{m,r,y}, \forall m \in \Omega^{\mathrm{HU}} r,y,d,t \tag{53}$$

- Spinning Reserve Requirements during Operation

Spinning reserve constraints represent the flexibility requirements during power system operation. Due to the uncertainties in the load and VRE output, the actual value may fluctuate greatly in a short time. Hence, the system must have sufficient spinning capacity to supplement potential power shortages. Here, we define the spinning capacity as the output increments generators and ESSs can provide within 10 minutes. The expression of spinning requirements is presented in (54).

$$\begin{aligned}\sum_{m\in\Omega^{\mathrm{GP}},\Omega^{\mathrm{ESS}}} P^{\mathrm{hot}}_{m,r,y,d,t} + \sum_{m\in\Omega_r^{\mathrm{DC,to}}} P^{\mathrm{hot}}_{m,r,y,d,t} \ge &\mathrm{hr}_r^{\mathrm{Load}} \cdot \left(L_{r,y,d,t} + \sum_{m\in\Omega_r^{\mathrm{DC,from}}} P^{\mathrm{hot}}_{m,r,y,d,t} \right) + \\ &\mathrm{hr}_r^{\mathrm{Wind}} \cdot \sum_{m\in\Omega^{\mathrm{Wind}}} P^{\mathrm{Wind}}_{m,r,y,d,t} + \mathrm{hr}_r^{\mathrm{PV}} \cdot \sum_{m\in\Omega^{\mathrm{PV}}} P^{\mathrm{PV}}_{m,r,y,d,t}, \forall r,d,y,t\end{aligned} \tag{54}$$

where $P^{\mathrm{hot}}_{m,r,y,d,t}$ denotes the spinning reserve capacities unit m in bus r can provide in time period t of representative day d at stage y. The spinning reserves are set to address fluctuations in load and variable renewable energy. The three terms on the right side present the spinning demand caused by load, wind and PV power. hr_r is the spinning factor, which is set to 5% according to planning criteria and grid codes in China[15]. The spinning capacities must cover potential shortages. The spinning reserves provided by various units are as follows:

$$0 \le P^{\mathrm{hot}}_{m,r,y,d,t} \le \min\left\{U^{\mathrm{on}}_{m,r,y,d,t} - P_{m,r,y,d,t},\ \mathrm{rp}_m \cdot U^{\mathrm{on}}_{m,r,y,d,t}\right\}, \forall m \in \left\{\Omega^{\mathrm{TU}}, \Omega^{\mathrm{NU}}\right\}, r,t,d,y \tag{55}$$

$$0 \le P^{\mathrm{hot}}_{m,r,y,d,t} \le \min\left\{U^{\mathrm{on}}_{m,r,y,d,t} - P^{\mathrm{net,CCS}}_{m,r,y,d,t},\ \mathrm{rp}_m \cdot U^{\mathrm{on}}_{m,r,y,d,t}\right\}, \forall m \in \left\{\Omega^{\mathrm{CCS}}\right\}, r,t,d,y \tag{56}$$

$$0 \le P^{\mathrm{hot}}_{m,r,y,d,t} \le U^{\mathrm{GP}}_{m,r,y} - P_{m,r,y,d,t}, \forall m \in \Omega^{\mathrm{HU}}, r,t,d,y \tag{57}$$

$$0 \le P^{\mathrm{hot}}_{m,r,y,d,t} \le \min\left\{U_{m,r,y} - P_{m,r,y,d,t},\ 6 \cdot S^{\mathrm{TES}}_{m,r,y,d,t} \cdot \eta^{\mathrm{PB}}_m\right\}, \forall m \in \Omega^{\mathrm{CSP}}, r,t,d,y \tag{58}$$

$$0 \le P^{\mathrm{hot}}_{m,r,y,d,t} \le \min\left\{U^{\mathrm{ESS}}_{m,r,y} - P^{\mathrm{dis}}_{m,r,y,d,t} + P^{\mathrm{ch}}_{m,r,y,d,t},\ 6 \cdot S^{\mathrm{ESS}}_{m,r,y,d,t} \cdot \eta^{\mathrm{ESS}}_m\right\}, \forall m \in \Omega^{\mathrm{ESS}}, r,t,d,y \tag{59}$$

$$0 \le P^{\mathrm{hot}}_{m,r,y,d,t} \le U^{\mathrm{DC}}_{m,y} - f^{\mathrm{DC,to}}_{m,y,d,t}, \forall m \in \Omega_r^{\mathrm{DC,to}}, r,t,d,y \tag{60}$$

where rp_m denotes the maximum ramp rate within 10 minutes for unit m. The term, $6 \cdot S^{\mathrm{ESS}}_{m,r,y,d,t} \cdot \eta^{\mathrm{ESS}}_m$ denotes the maximum power that ESSs can provide by discharging the whole energy storage within 10 minutes. $P^{\mathrm{hot}}_{m,r,y,d,t}$ in Eq.(60) denotes the spinning reverse provided to the receiving end by DC transmission lines. The upper boundary of $P^{\mathrm{hot}}_{m,r,y,d,t}$ here is equal to the difference between the rated capacity and the current power flow. The spinning reverse demands are also transferred to the sending end as in Eq.(54). In our model, the spinning capacities indicate the capacities that flexibility units are able to be provided within 10 minutes. The dispatch of the AC line power flow is difficult to achieve within this time scale because it relies on the adjustment of the generator output and switching operation. Hence, AC lines are not modelled in the constraints of "Spinning Reserve Requirements".

- Minimum System Inertia Limits

As VRE penetration increases, the frequency stability characteristics of the power system change dramatically. This change comes from the characteristics of low inertia and the weak frequency regulation ability of wind and PV generators. With the installed capacities of conventional power sources decreasing, the level of synchronization inertia provided by the synchronous machine will continue to decrease. The reduction in inertia will directly affect the rate of change of frequency (RoCoF) and the minimum frequency when system faults occur. The blackout that occurred in the UK in August 2019 was caused by an excessively large RoCoF (0.125/Hz) after the withdrawal of wind farms at the initial stage of failure[16]. Low inertia is a characteristic problem of high renewable energy penetration systems[17]. Here, this model introduces the minimum system

inertia limits to guarantee frequency stability with high renewable penetration. The expressions of minimum system inertia requirements are presented in (61)-(63).

$$\sum_m H_{m,r,y,d,t} \geq \alpha \cdot h_0 \cdot L_{r,y,d,t}, \forall r, y, d, t \quad (61)$$

$$H_{m,r,y,d,t} = h_m \cdot U^{\text{on}}_{m,r,y,d,t}, \forall m \in \left\{\Omega^{\text{TU}}, \Omega^{\text{NU}}, \Omega^{\text{CCS}}\right\}, r, t, d, y \quad (62)$$

$$H_{m,r,y,d,t} = h_m \cdot U_{m,r,y}, \forall m \in \left\{\Omega^{\text{Hydro}}, \Omega^{\text{ESS}}, \Omega^{\text{CSP}}\right\}, r, t, d, y \quad (63)$$

where the coefficient h_m denotes the inertia constant of unit m. The total inertia that can be provided by thermal units and nuclear units is equal to the product of the online capacity and inertia constants. The inertia from hydro, ESS and CSP is equal to the product of installed capacity and their inertia constants because of their fast grid-connection ability. The term on the right-hand side in (61) denotes the minimum inertia required. $L_{r,y,d,t}$ represents the load of the current period t. h_0 denotes the inertia level for China's current power system. α is a constant reflecting the tolerance of the system inertia drop, which is set to 0.7 in the base case of this paper. This value means the system operators allow the system inertia to decrease by 30% from the current level. The specific values of the parameters for generation units, ESSs and lines are presented in Supplementary Tables 14 to 20.

The inertia of the power system mainly comes from the rotating parts of the local generators or the virtual inertia provided by the energy storage systems (ESSs). The long-distance transmission effect of the system inertia is still unclear and belongs to the field of power system transient analysis, which is beyond the scope of this paper. Therefore, we assume that the transmission network cannot transfer inertia between provinces on the transient time scale. In other words, the inertia cannot be transmitted through a long-distance transmission power system during the transient process. We consider the inertia constraints for each province individually for system transient stability.

Supplementary Note 1.5 Solving Method and Code Implementation

The GTEP model is a large-scale linear programming problem with multiple coupled periods. We apply Gurobi 9.1, an advanced off-the-shelf optimization solver, to solve the GTEP model. Classical linear programming methods include the simplex method and the barrier method. Due to the characteristics of multiple coupled periods, the feasible region of the GTEP model contains massive extreme points (vertices). The simplex method may fail in such cases; hence, we select the barrier method, whose performance is less sensitive to the number of extreme points[18]. The tolerance gap is set to 0.0001. The model and case studies are implemented using MATLAB on a standard workstation with an Intel(R) Core(TM) i9-10900K@3.70 GHz CPU and with 64.0 GB of RAM. YALMIP, a toolbox for modelling and optimization in MATLAB, is applied to construct the GTEP model[19]. The time consumption for solving the model is approximately one hour but varies slightly with different case settings. The memory occupied during the solution process is roughly 15-20 GB.

Supplementary Note 2 Piecewise Supply Curves Calculation

The LCOE of VRE increases remarkably due to the decrease in capacity factors and the increase in the difficulty of construction and grid integration with the growth of installed capacity. This variation must be considered in the model. Without considering the spatial distribution of LCOE, the cost will be underestimated by 2.2 CNY¢/kWh, as discussed in the main paper. We assess the LCOE for every 500m×500m plot in each province based on the GREAN dataset and the method described in Fig. 6. Consequently, the provincial VRE supply curves can be obtained, as shown in Supplementary Figs. 7 and 8. Supply curves characterize the quantity, quality, and cost of renewable resources. The reason for the change in LCOE is due mainly to the differences in capacity factors and grid-connection costs. According to our assumptions, only one aggregated unit is considered in each province to simplify the model. Naturally, one unit can correspond to only one capacity factor, which is set as the average value over the province in this paper. Hence, the supply curves cannot be integrated into the GTEP model, an optimization problem directly where the total generation costs consist of operating costs, maintenance costs and capital costs. Here, we convert the supply curves of LCOE into supply curves of capital cost considering no fuel costs and low proportion of maintenance costs for wind and PV power. The calculation is as follows:

$$I_{m,r}^{\text{INV,type}} = 8760 \cdot I_{m,r}^{\text{LCOE,type}} \cdot \text{cf}_{\text{m,r}}, \quad \text{type} \in \{\text{VRE}\} \tag{64}$$

where $I_{m,r}^{\text{LCOE,type}}$ denotes the LCOE for unit m in province r and $\text{cf}_{\text{m,r}}$ denotes the average capacity factor for unit m in province r. Thus, we can integrate supply curves into the GTEP model through the dynamic change in capital cost with the installed capacity.

The dynamic change in capital cost is considered in the GTEP model in a piecewise manner. The GTEP model is a linear programming model; hence, the non-linear capital cost curve cannot be integrated directly. Here, we split the curve into a small number of segments. Each segment corresponds to the capital cost of a certain value. The width of the segments represents the capacity than can be installed with the corresponding capital costs. Essentially, the split uses a piecewise linear function to fit the capital cost curve, which is an optimization problem of minimizing the square error. We applied the open-source PWLF function package implemented in python to fit the curve. The differential evolution optimization algorithm, a popular heuristic algorithm, is used in this package to find the best location for the user-defined number of line segments, which is set to seven. The width of the segments $\overline{U}_s^{\text{type}}$ and their corresponding capital cost $I_{m,r,s}^{\text{LCOE,type}}$ are the results of the fitting algorithm. Their roles in the GTEP model are detailed in (3) and (15)-(17). Supplementary Fig. 1(a) illustrates the fitting results of wind power capital costs in Fujian province. The installed capacity is normalized by dividing by the maximum developable capacity, and the capital cost per kW is normalized by dividing by the average capital cost. Notably, the capital cost curve is used to calculate the total capital cost, i.e., the integration of the capital cost curve to installed capacity. The fitting of the piecewise linearized function on total capital cost is shown in Supplementary Fig. 1(b).

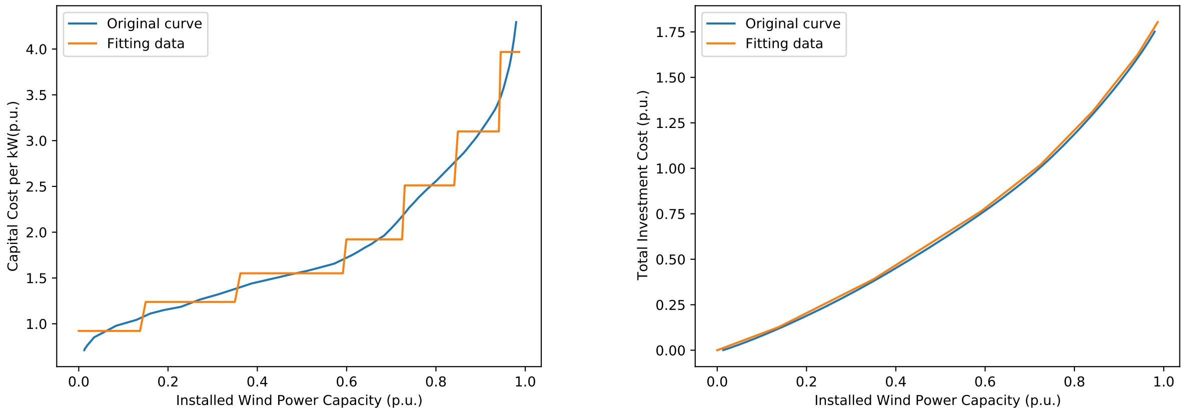

(a) Fitting results of wind power capital costs in Fujian province (b) Fitting results of total wind capital costs in Fujian province

Supplementary Figure 1. Illustration of piecewise linear fitting for VRE supply curve

The fact that VRE capital costs per kW account for a considerable fraction of the supply costs shows the necessity of considering the supply curves for each province in detail. For comparison, the supply curve is set to 1, a horizontal straight line,

to represent the case where the spatial distribution of LCOE is not considered. Specifically, the capital cost per kW $I^{\mathrm{INV,type}}_{m,r,s}$ is set to 1 for each segment. Absent consideration of the spatial distribution of LCOE, the costs in 2050 will be underestimated by 2.2 CNY¢/kWh.

Supplementary Note 3 Calculation of Electricity Supply Costs and Carbon Mitigation Costs

Supplementary Note 3.1 Electricity Supply Costs

The electricity supply cost is the average cost the power system must pay to supply per kWh of electricity load demand. For a single stage, this value is the ratio of the total cost caused by power generation and transmission to the load demand in the stage, as follows:

$$c_y = \frac{c_y^{\text{total}}}{\sum\limits_{r,t}\sum\limits_{d}\varphi_d D_{r,y,d,t}} \forall y \tag{65}$$

where c_y denotes the electricity supply cost at stage y. c_y^{total} denotes the total cost caused by power generation and transmission in year y. $\sum\limits_{r,t}\sum\limits_{d}\varphi_d D_{r,y,d,t}$ denotes the total electricity load demand where φ_d is the number of duration days of representative day d within one stage. The total cost c_y^{total} consists of three parts: operating costs c_y^{OP}, maintenance costs c_y^{MAT} and capital costs c_y^{CAP}. The expression is as follows:

$$c_y^{\text{total}} = c_y^{\text{MAT}} + c_y^{\text{OP}} + c_y^{\text{CAP}} \tag{66}$$

The electricity supply costs correspond to future years. Hence, it is not necessary to convert the costs into present values, as in (1). The calculation of c_y^{MAT} and c_y^{CAP} is the same as (4) - (9) because they are originally calculated for a single stage. Note that the definition of capital costs c_y^{CAP} is different from C_y^{INV} in the objective function shown in (2). C_y^{INV} represents the capital cost in stage y. For electricity supply costs, the capital costs are the annualized capital costs of all devices existing in the power system at this stage. The calculation of c_y^{CAP} is as follows:

$$c_y^{\text{CAP,type}} = \sum_r \sum_{m\in\Omega^{\text{type}}} \sum_{y_i=0}^{y} \frac{i}{1-(1+i)^{-Y_m}} I_{m,y_i}^{\text{INV,type}} U_{m,r,y,y_i}^{\text{type}}, \quad \forall \text{type} \in \{\text{GP}\backslash\text{VRE}, \text{ESS}, \text{TL}\} \tag{67}$$

$$c_y^{\text{CAP}} = \sum_{\text{type}} c_y^{\text{CAP,type}} \tag{68}$$

where $U_{m,r,y,y_i}^{\text{type}}$ denotes the remaining capacity of unit m that is invested during stage y_i of stage y in province r. When y_i is equal to zero, $U_{m,r,y,0}^{\text{type}}$ denotes the remaining capacity of unit m that originally exists in the system at stage y. The planning period in GTEP model is from 2020 to 2050, and the variation in capital costs before 2020 is not considered in the calculation. We assume that the capital costs of existing devices are equal to the value in 2020. For wind and PV units, whose developing potential is split into several segments, the expression is modified as follows:

$$c_y^{\text{CAP,type}} = \sum_r \sum_{m\in\Omega^{\text{type}}} \sum_s \sum_{y_i=0}^{y} \frac{i}{1-(1+i)^{-Y_m}} I_{m,y_i}^{\text{INV,type}} U_{m,r,y,s,y_i}^{\text{type}}, \quad \forall \text{type} \in \{\text{VRE}\} \tag{69}$$

Supplementary Note 3.2 Marginal Carbon Prices

Marginal carbon prices are the cost increase per tonne of carbon emission reduction under certain emission limits. They represent the sensitivity of the objective function to carbon emission limits. From the perspective of an optimization problem, the marginal carbon prices are the shadow prices of the carbon emission limit constraints (23). Shadow prices reflect the scarcity of related resources[20]. There are alternative names for shadow prices, such as optimal dual variable values or optimal Lagrange multipliers. By solving the GTEP model, a linear programming problem, the shadow prices can be obtained directly because they are necessary intermediate parameters during the barrier algorithm[21]. When the carbon taxes are set to the value of marginal carbon prices as a penalty term, the carbon emission results would naturally be the constrained value even without the forced carbon emission constraint (23) in the model.

However, the objective function is the present value at the end of 2020. The original shadow prices are supposed to be converted to a future value in the corresponding stage. The expressions are as follows:

$$\lambda_y = (1+i)^{5y} \lambda_y^{\text{raw}} \tag{70}$$

where λ_y^{raw} denotes the original shadow price provided by the solver for carbon emission limits at stage y. i is the discount rate, and λ_y is the converted margin prices in future values.

Supplementary Note 3.3 Average Carbon Mitigation Costs

Average carbon mitigation costs are the additional costs per tonne of carbon emission between two scenarios. This value is numerically equal to the ratio of the difference between the total cost of the two scenarios and the difference between the carbon emission budget. The expression is as follows:

$$\overline{\lambda}_{n,m} = \frac{C_n - C_m}{E_m - E_n} \tag{71}$$

where $\overline{\lambda}_{n,m}$ denotes the average carbon mitigation costs between scenarios n and m. C_n and E_n are the total costs and carbon emission budget during the planning period for scenarios n, respectively.

Note that there is no direct relationship between marginal carbon prices and average carbon mitigation costs since they are calculated via different methods, as discussed above. Roughly speaking, if we regard the total costs as a function of the carbon emission budget, marginal carbon prices are the gradient of the function at a certain point. Thus, the average carbon mitigation costs are the average gradient of the function over a certain interval or between two points.

Supplementary Note 4 Sensitivity Analysis Setting

Various uncertainties impact the final supply cost results. We analyse the sensitivities of the electricity supply costs to five factors (RE capital cost, RE unit production capacity, transmission limits, reserve and inertia requirements, and load growth rates) under the CN2050 scenario. A low and high scenario is considered for each factor. Different scenarios correspond to different parameters in the objective functions or right-hand side vector of the optimization model. We scan the parameters within the given intervals with five intermediate scenarios sampled evenly. The specific settings for the five uncertain factors are as follows.

- RE and BESS Capital Costs

The RE and BESS capital cost (per kW) projection in the GTEP model refers to NREL's annual technology baseline (ATB) model results published in 2021[22], which contains three trajectories: conservative, moderate, and advanced. The conservative trajectory is set as the high-cost case, and the advanced trajectory is set as the low-cost case. The average capital costs in the different five-year stages are presented in Fig. 2. The capital costs are normalized by the capital costs in 2020. Capital costs of battery energy storage (BESS) are also included. The capital cost uncertainty of pumped hydro storage (PHS) is not considered in the sensitivity analysis because of its mature technical level.

- RE and BESS Manufacturing Capability

RE and ESS manufacturing capability denotes the maximum newly installed capacity at each five-year stage, which is $\Delta\overline{\mathrm{Ui}}^{type}_{m,r,y}$ in (12). The average growth capacities of wind and PV in the past five years are 30.0 GW and 41.8 GW annually. It is reported that approximately 100 GW VRE units have started construction at the end of 2021[23]. Hence, we set the maximum VRE manufacturing capability in the first planning stage (2021-2025) to 100 GW per year for the base case. We consulted several experts from the China Electric Power Planning and Engineering Institute and set the future maximum VRE manufacturing capability at about 260 GW per year. We assumed that VRE manufacturing capability grows linearly and reaches a maximum around 2035. The maximum manufacturing capability for all kinds of RE units at each stage is presented in Supplementary Fig. 3. The manufacturing capability of pumped hydro storage (PHS) is also not considered in the sensitivity analysis because of its mature technical level.

- Load Growth Rates

We assume that load demand would fluctuate 5% compared with the baseline scenario, i.e., the CN2050 scenario. The high scenario assumes that demand increases linearly 5% until 2050. The low scenario assumes that demand decreases linearly 5% until 2050. The total electricity energy demands under different scenarios are presented in Table 1.

- Security and Inertia Requirements

Three kinds of secure reserve constraints are considered in the GTEP model in both the planning and operation periods. The three constraints are the power reserve requirements (24), hot reserve requirements(54), and minimum system inertia limits(61). To analyse the impacts of security requirements on electricity supply costs, we set different secure requirement levels with different parameter settings on rs, hr, and α, as shown in Table 2. The parameter setting of the low scenario is equivalent to that of no security and inertia constraint. The parameters in different provinces are set to the same values, except rs under the base case. The value of 0.13 presented in 2 is the average value under the base case. The specific power reserve rate for each province is provided in [24].

- Transmission Limits

The impacts of the transmission capacity on the electricity supply costs are calculated by adjusting the upper limits on each transmission corridor. The low scenario assumes the candidate transmission limits scale down by 50% compared with the base case. The high scenario assumes the candidate transmission limits scale up by 100% compared with the base case. The total transmission limits of candidate AC and DC lines under different scenarios are presented in Table 3.

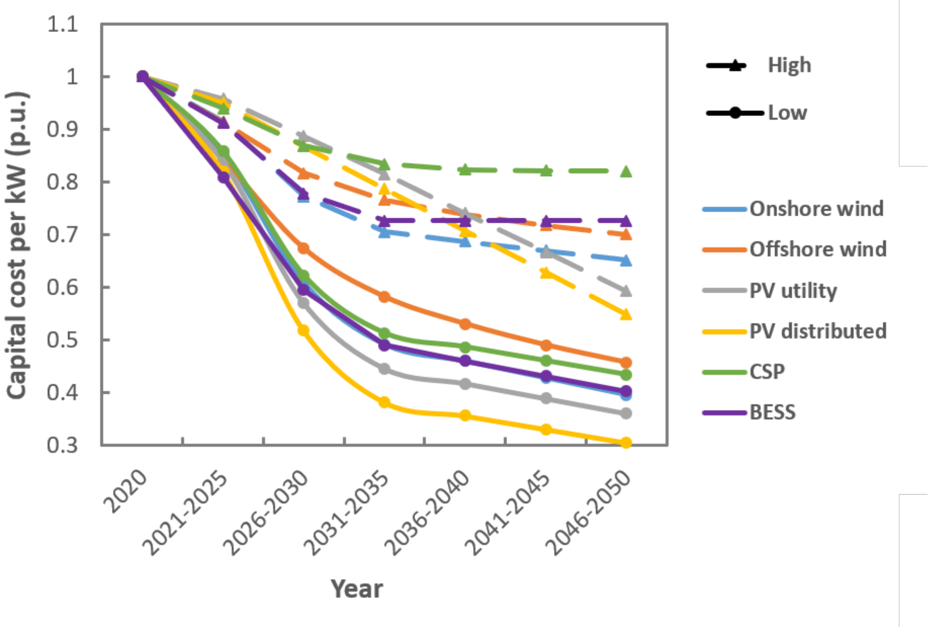

Supplementary Figure 2. **Capital cost projection of RE generation technologies for the 30 years.**

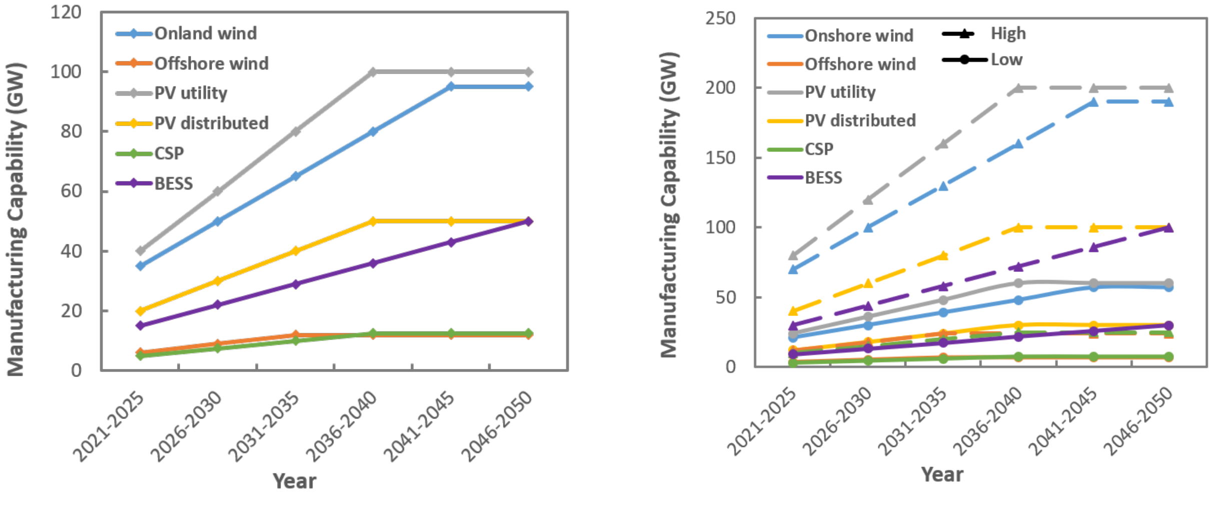

(a) Manufacturing capability settings in the CN2050 base scenarios (b) Sensitivity analysis settings on the manufacturing capability

Supplementary Figure 3. **Average manufacturing capability at each five-year stage.**

Supplementary Table 1. **Total electricity energy demands under different scenarios(TWh)**

	2020	2025	2030	2035	2040	2045	2050
Low	7511	8825.5	10459.5	11628	12457.7	13287.3	14117
Base	7511	9290	11010	12240	13113.3	13986.7	14860
High	7511	9754.5	11560.5	12852	13769	14686	15603

Supplementary Table 2. **Secure requirement level setting under different scenarios**

	rs	hr^{Load}	hr^{Wind}	hr^{PV}	α
Low	-1	0	0	0	0
Base	0.13	0.05	0.05	0.05	0.7
High	0.2	0.15	0.15	0.15	1

Supplementary Table 3. **Total transmission limits of AC and DC candidate lines under different scenarios (GW)**

	AC lines	DC lines
Low	460.3	284
Base	920.6	568
High	1841.2	1136

Supplementary Note 5 Renewable energy potential and supply curves in China

China's wind and photovoltaics (PV) energy potential is assessed based on the Global Renewable-energy Exploitation Analysis (GREAN) database. The total economic potential for wind and solar PV is 7.2 TW and 128.1 TW, respectively. The total energy potential is 200.9 PWh per year, which is 13.5 times China's maximum projected electricity demand in 2050 of 14.9 PWh yr^{-1}. Table 4 presents the VRE potential and capacity factor distribution in China projected by this study. Due to the low construction requirements of PV plants for site and weather conditions, the economic potential for PV power far exceeds that of wind power. It is theoretically feasible to achieve carbon neutrality in China's power sector via high RE penetration.

Although the total amount is fairly abundant, the spatial distribution of RE potential and the corresponding LCOE are not uniform and are highly mismatched with the electricity demand. RE-rich areas for both wind and PV are located mainly in Northern China and Northwestern China, while the load centres are in coastal areas. Specifically, the four provinces of Xinjiang, Qinghai, Gansu, and Inner Mongolia account for 75.5% RE potential with high capacity factors but account for only 11% of the electricity consumption. Numerically, the RE economic potential in the load centres can locally cover a considerable fraction of the demand; however, the capacity factors for RE units are relatively low, which leads to high LCOE. Hence, the supply curves across the whole country must be considered when optimizing RE investments and regional network connections.

The RE potential varies greatly not only between provinces but also in different areas within a single province. Supplementary Fig. 7 and Supplementary Fig. 8 present the spatial VRE capacity factor distribution and regional LCOE in each province across the Chinese mainland. The average wind and PV LCOE in eastern provinces are higher than those in western provinces by 6.46 CNY¢/kWh (26.0%) and 2.95 CNY¢/kWh (16.1%), respectively. Along with the accelerating development of RE, the LCOE increases remarkably due to the decrease in capacity factors and the increase in the difficulty of construction and grid integration. This feature is particularly obvious for wind power. The cost distribution for onshore wind shows a "fat tail" pattern, which means the costs are distributed within a large range. The LCOE ranges from 17.5.7-54.8 CNY¢/kWh in different regions (95% confidence level), with a nationwide average of 26.7 CNY¢/kWh. The LCOE of PV distribution is relatively concentrated and varies from 11.7 to 31.5 CNY¢/kWh (95% confidence level), with a nationwide average of 19.5 CNY¢/kWh. The RE potential and supply curves in each province determine the development sequence and spatial distribution of renewable energy units, which further determines the future power system morphology under carbon neutrality targets. During the transition, the cost increase caused by the supply curves and the cost decrease brought about by technological advancements together drive the electricity supply cost.

Supplementary Table 4. VRE potential and capacity factor distribution in China projected by this study

Region	Onshore Wind		Solar PV	
	Total Potential (TW)	Average Capacity Factors	Total Potential (TW)	Average Capacity Factors
North China (N)	3.35	0.268	30.36	0.184
Northeast China (NE)	0.68	0.252	3.31	0.160
East China (E)	0.22	0.221	2.64	0.121
Central China (C)	0.27	0.188	4.07	0.116
South China (S)	0.28	0.224	6.56	0.136
Southwest China (SW)	0.02	0.207	7.40	0.179
Southeast China (SE)	2.35	0.251	73.75	0.164
China	7.17	0.256	128.09	0.167

Supplementary Note 6 Comparison with similar existing literature

The main new features of the GTEP model in our paper compared with existing literature are threefold:

- **The supply curves of wind and PV power are integrated into the GTEP model in a piecewise manner to present the impacts of VRE resource spatial distribution.** Without considering the spatial distribution of LCOE, the cost will be underestimated by 2.2 CNY¢/kWh according to our study. The VRE supply curves of each province in China are shown in Supplementary Fig. 7 and Supplementary Fig. 8. Details on the piecewise calculation method are presented in Supplementary Note 2.

- **The model includes three kinds of power system security and stability constraints: power reserve limits, spinning reserve requirements, and minimum system inertia limits.** These three constraints respectively correspond to the security challenges of high RE penetrated power systems in the time scale of planning, operation, and transient stability. Such constraints determine the additional flexible resources that are required to accommodate the increasing RE. Their mathematical expressions are presented in Eq.(24) and Eq.(55)-Eq. (63) of SI. Few existing studies model minimum system inertia limits in the expansion model, which would underestimate the electricity supply costs.

- **We project local network expansion within provinces based on the historical data and the planning results of the GTEP model.** Meanwhile, we modify the local grid investment according to the changes in the capacity factors of the local capacity mix. The projection method is introduced in the Method section. The projection results for the local grids are shown in Supplementary Table 7 and Supplementary Table 8. The electricity supply costs caused by local network investment and maintenance are 11.9 CNY¢/kWh (20.5%) in 2050 under the CN2050 scenario. Therefore, the capital costs and maintenance costs of the transmission and distribution system within each province account for a considerable portion of the total electricity supply cost, which is not considered in detail in previous studies.

We compared our model with previous studies on the low-carbon transition of China in terms of other modelling details as shown in Supplementary Table 5. In addition to the above three main features, our model provides advantages related to power equipment modelling, a high temporal resolution, and a long planning period duration. He et al.[3] proposed a "SwitchChina" model to study the impacts of rapid RE cost decrease on low-carbon transition. The model has made progress in comprehensive national power system planning. But minimum system inertia limits, the critical challenges brought by high RE penetration are not considered. Due to its early publication time, some important new elements such as CCS and biomass energy did not participate in the low-carbon transition, either. To reduce the calculation burden, the minimum time resolution in "SwitchChina" model is set to six hours while the minimum time resolution is one hour in our model. This will impact the characterization of wind and PV intermittency during the operating simulation. Please see other model feature differences in Supplementary Table 5.

It is hard to compare the cost results with existing articles directly because most studies have different target years and model settings. Some studies focus on the power system morphology and the results of electricity supply costs are not even discussed[12,25]. The target years of 3 and 26 are 2030 and 2035, respectively. We pick two scenarios from 3 and 26 where the results are similar to ours for comparison as shown in Supplementary Table 5. In 3, the electricity supply cost is 8.91 USD¢/kWh in 2030 with 80% carbon emission reduction. In 26, the electricity supply cost is 8.69 USD¢/kWh in 2035 with 75% RE penetration. It can be inferred that the electricity supply costs based on 3 and 26 will also be lower than that of our model under the scenario of carbon neutrality in 2050 because they require similar carbon emission targets to be reached earlier than our CN2050 scenario. The differences mainly come from the above three new features of our model mentioned above.

Chen et al.[27] proposed a single-period investment planning model for China's power system where the RE penetration is forced in the target year of 2050. The RE penetration in 27 only includes wind, PV, and hydro power. The electricity supply cost is 2.72 USD¢/kWh under the base case with the RE penetration of 80%. The cost is much lower than our results (8.39 USD¢/kWh) in the CN2050 scenario whose RE penetration is 86.2%. We summarize the reasons for the lower costs in 27 as follows:

- Inner-provincial transmission network development and power losses caused by transmission are not considered in 27.

- Minimum system inertia limits are not considered in 27. Moreover, the model does not set redundant power reserves as required by engineering practice (i.e. the power reserve rate *rs* in Eq. (24) of SI is set to 0.

- The VRE supply curves are not integrated into the model. In other words, the investment costs of all wind farms in each province are assumed to be the same in 27.

- The expansion model in 27 is single-period which means no investment decision or carbon emission limit in the key intermediate years is considered. Thus, the change process of RE unit capital costs and manufacturing capability limits

are not modelled. The capital costs are calculated directly based on the value in the target year. However, our GTEP model is dynamic and multi-period.

- The LCOE of offshore wind power in 27 is too low, which is lower than the onshore wind power. The setting is unexpected and difference with most recent predictions[22, 28–30].

- Carbon neutrality is not fully achieved in the 80% RE penetrated scenario of 27 since the capacity mix retains about 1000 GW coal power. The less stringent transition goal leads to lower electricity supply costs.

Hence, simply estimating electricity supply costs based on only the power balance or the single-period model will result in underestimation of power system decarbonization costs. In particular, a fuller cost accounting must take stock of important practical considerations by integrating VRE supply curves into models, considering operational security concerns, projecting developments of the local network, and high time resolution modelling over the planning periods.

Supplementary Table 5. Comparison with existing articles about the low-carbon transition in China

Model feature		This paper	[3]	[26]	[27]	[12]	[25]
Base year		2020	2015	2020	2019	2016	2018
Target year		2050	2030	2035	2050	2030	2030
Number of planning periods		6	3	1	1	14	1
Number of typical days		12	24	365	365	4	4
Minimum time resolution		one hour	six hours	one hour	one hour	one hour	one hour
Region of planning		Mainland China	Mainland China	Northwest China	Mainland China without Tibet	Mainland China	Mainland China
Generation	Onshore wind	Yes	Yes. But no distinction.	Yes	Yes	Yes. But no distinction.	Yes. But no distinction.
	Offshore wind	Yes		No	Yes		
	PV utility	Yes	Yes. But no distinction.	Yes. But no distinction.	Yes. But no distinction.	Yes. But no distinction.	Yes. But no distinction.
	PV distributed	Yes					
	CSP	Yes	No	No	No	No	No
	Hydro	Yes	Yes	Yes	Yes	Yes	No
	Nuclear	Yes	Yes	No	Yes	Yes	Yes
	Biomass	Yes	No	No	No	No	No
	Coal	Yes	Yes	Yes	Yes	Yes	Yes
	Gas	Yes	Yes	Yes	Yes	Yes	Yes
	Biomass-ccs	Yes	No	No	No	No	No
	Coal-ccs	Yes	No	No	Yes	No	No
	Gas-ccs	Yes	No	No	No	No	No
	ESSs	Yes	Yes	Yes	Yes	No	Yes
Transmission	AC lines	Yes	Yes. But no distinction.	No	Yes	No	Yes. But no distinction.
	DC lines	Yes		No	Yes	No	
Are supply curves of VRE considered?		Yes	No	No	No	No	No
Are security constraints considered?	Power Reserve	Yes	Yes	Yes	Yes	No	No
	Spinning Reserve	Yes	Yes	Yes	Yes	No	No
	Minimum Inetia	Yes	No	No	No	No	No
Is the projection of the local grids expansion considered?		Yes	No	No	No	No	No
Electricity supply costs in the target year(USD¢/kWh)		8.39 (CN2050)	8.91 (C80)	8.69 (75% RE penetration)	2.72 (80% RE penetration)	N/A	N/A

Supplementary Figures

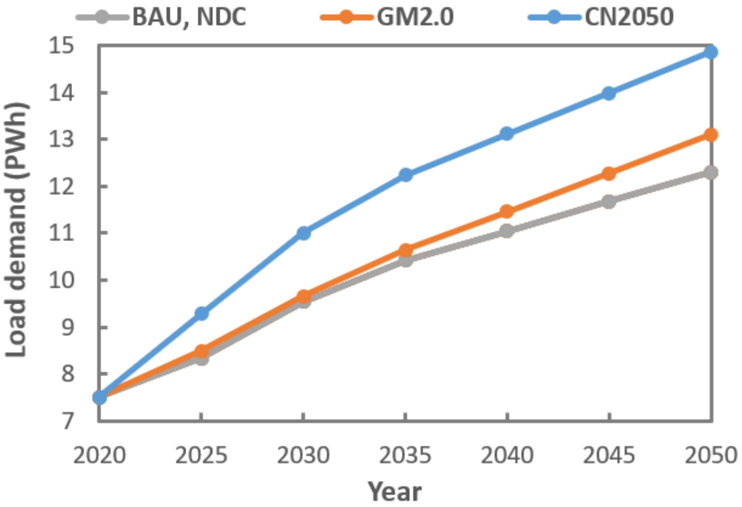

Supplementary Figure 4. **The electricity load demands under different scenarios**

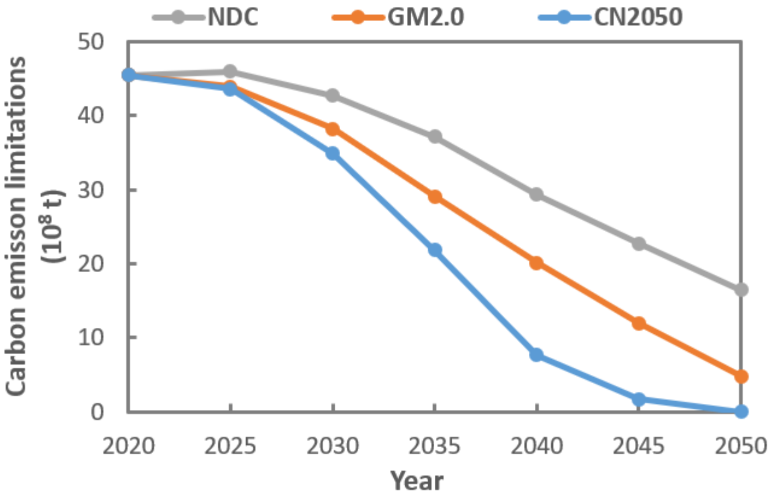

Supplementary Figure 5. **The carbon emission limit trajectories under different scenarios**

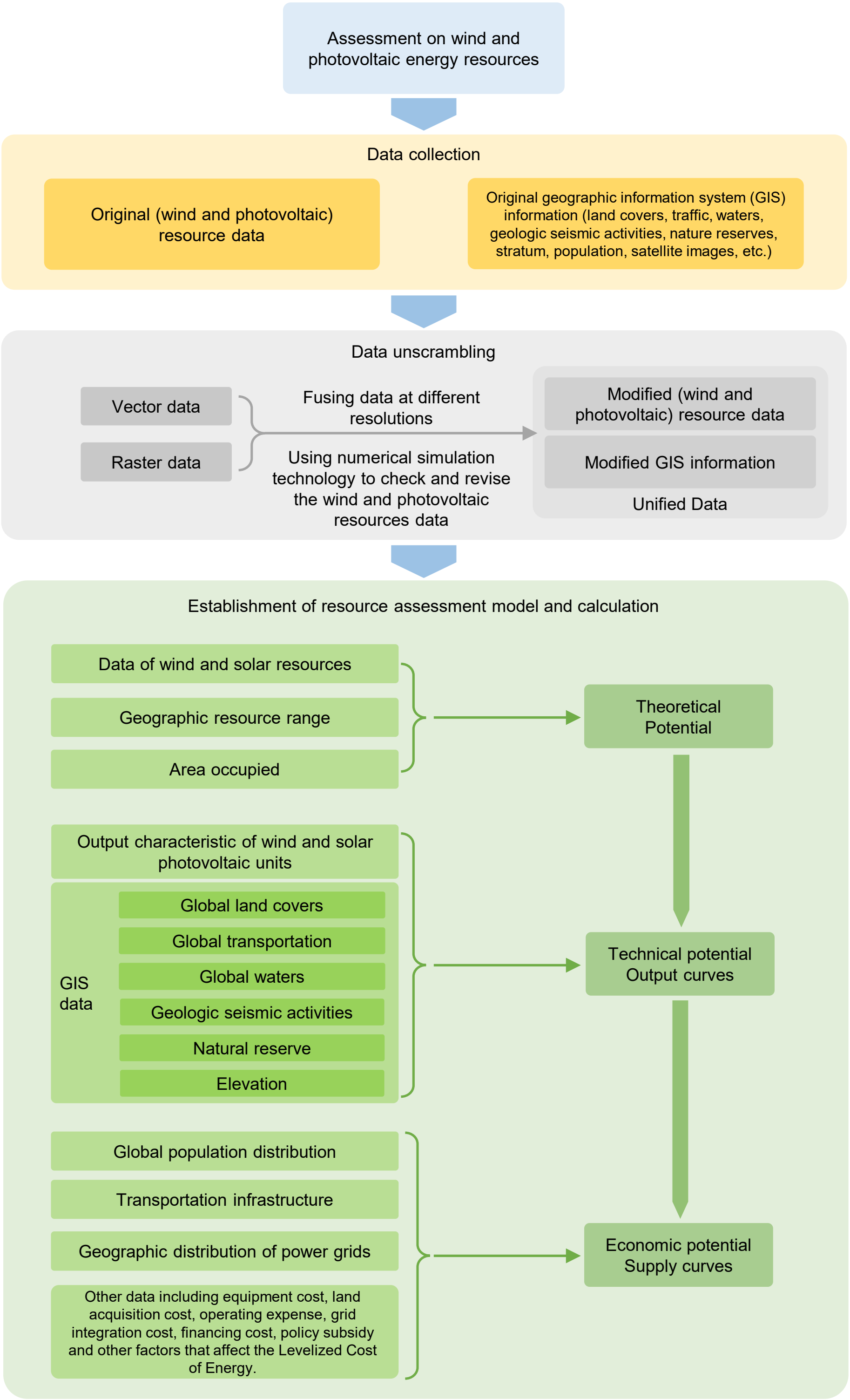

Supplementary Figure 6. Technical Procedure for GREAN Platform on VRE Resources Assessment

Supplementary Figure 6 shows the evaluation process of GREAN platform's for China's renewable energy potentials and supply curves. The detailed explanation is provided in Supplementary Ref. 31. The data sources of GREAN platform are described in Supplementary Tables 11 - 13.

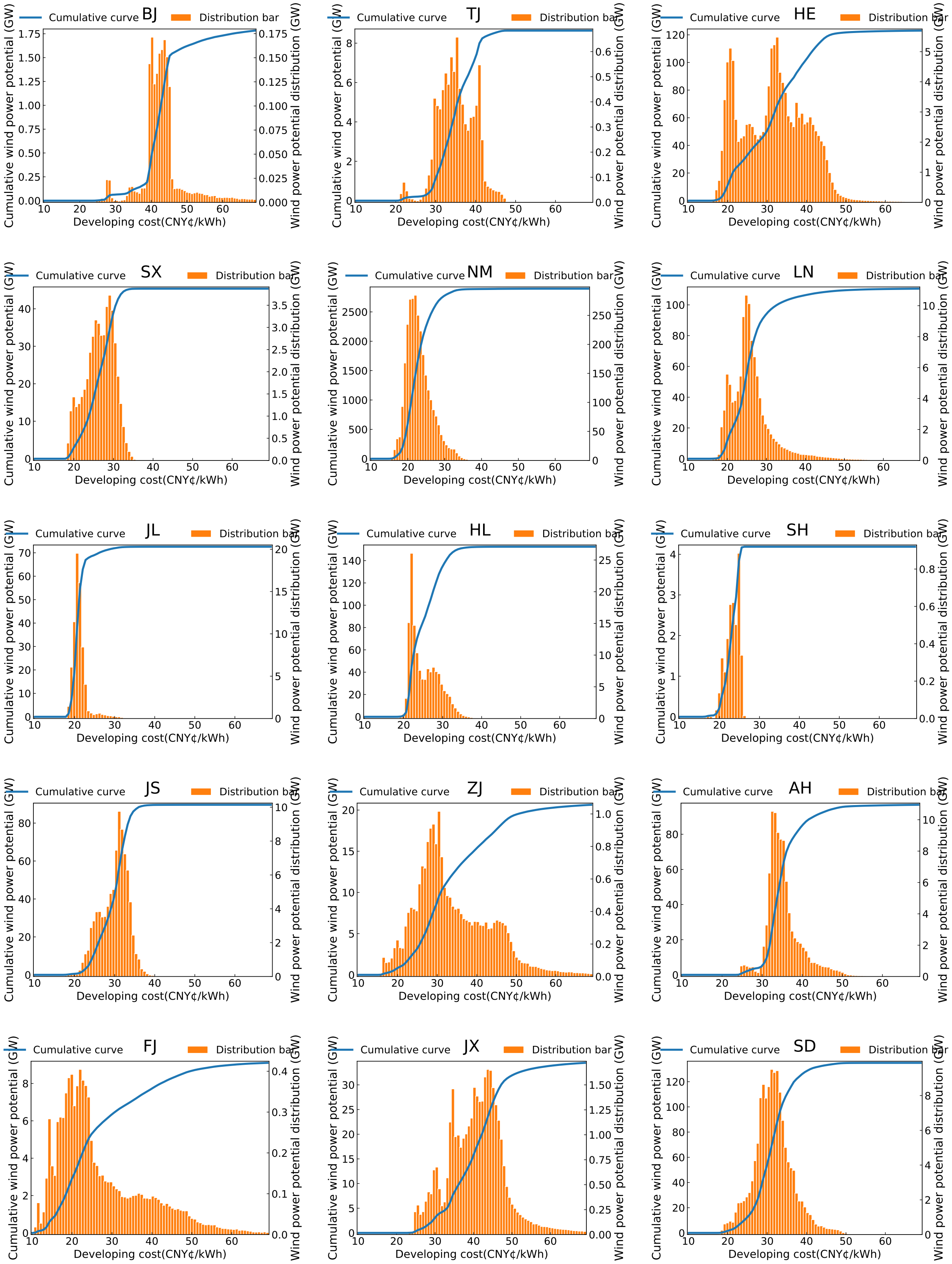
BJ
TJ
HE
SX
NM
LN
JL
HL
SH
JS
ZJ
AH
FJ
JX
SD
Cumulative curve
Distribution bar
Cumulative wind power potential (GW)
Wind power potential distribution (GW)
Developing cost(CNY¢/kWh)

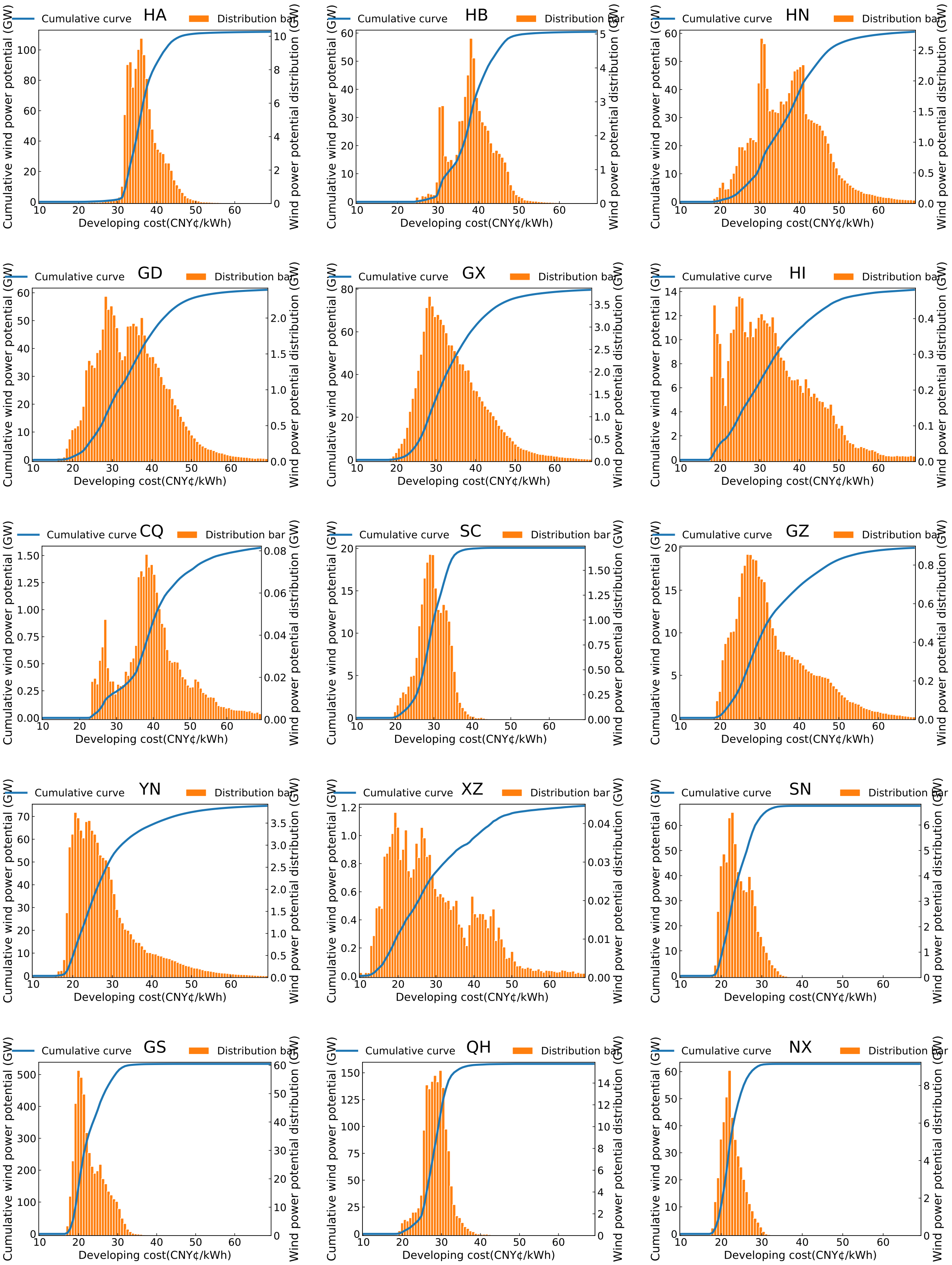
HA
HB
HN
GD
GX
HI
CQ
SC
GZ
YN
XZ
SN
GS
QH
NX
Cumulative curve
Distribution bar
Cumulative wind power potential (GW)
Wind power potential distribution (GW)
Developing cost(CNY¢/kWh)

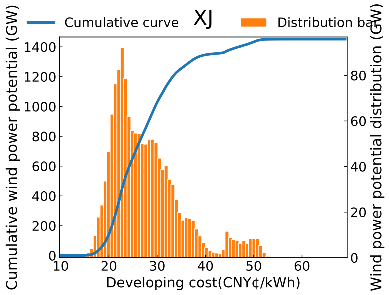

Supplementary Figure 7. **Supply curves of wind power in each province**

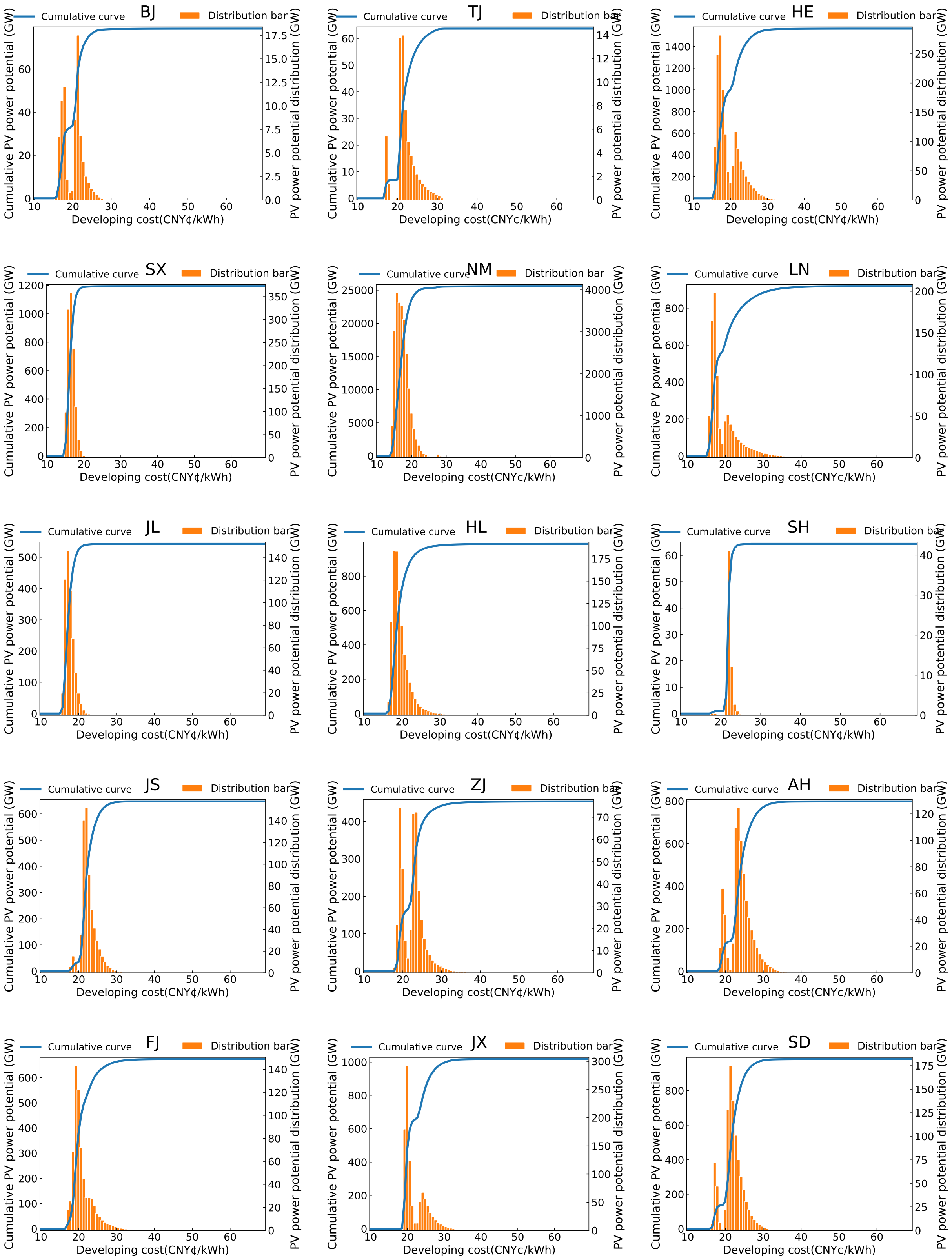
BJ
TJ
HE
SX
NM
LN
JL
HL
SH
JS
ZJ
AH
FJ
JX
SD
Cumulative curve
Distribution bar
Cumulative PV power potential (GW)
PV power potential distribution (GW)
Developing cost(CNY¢/kWh)

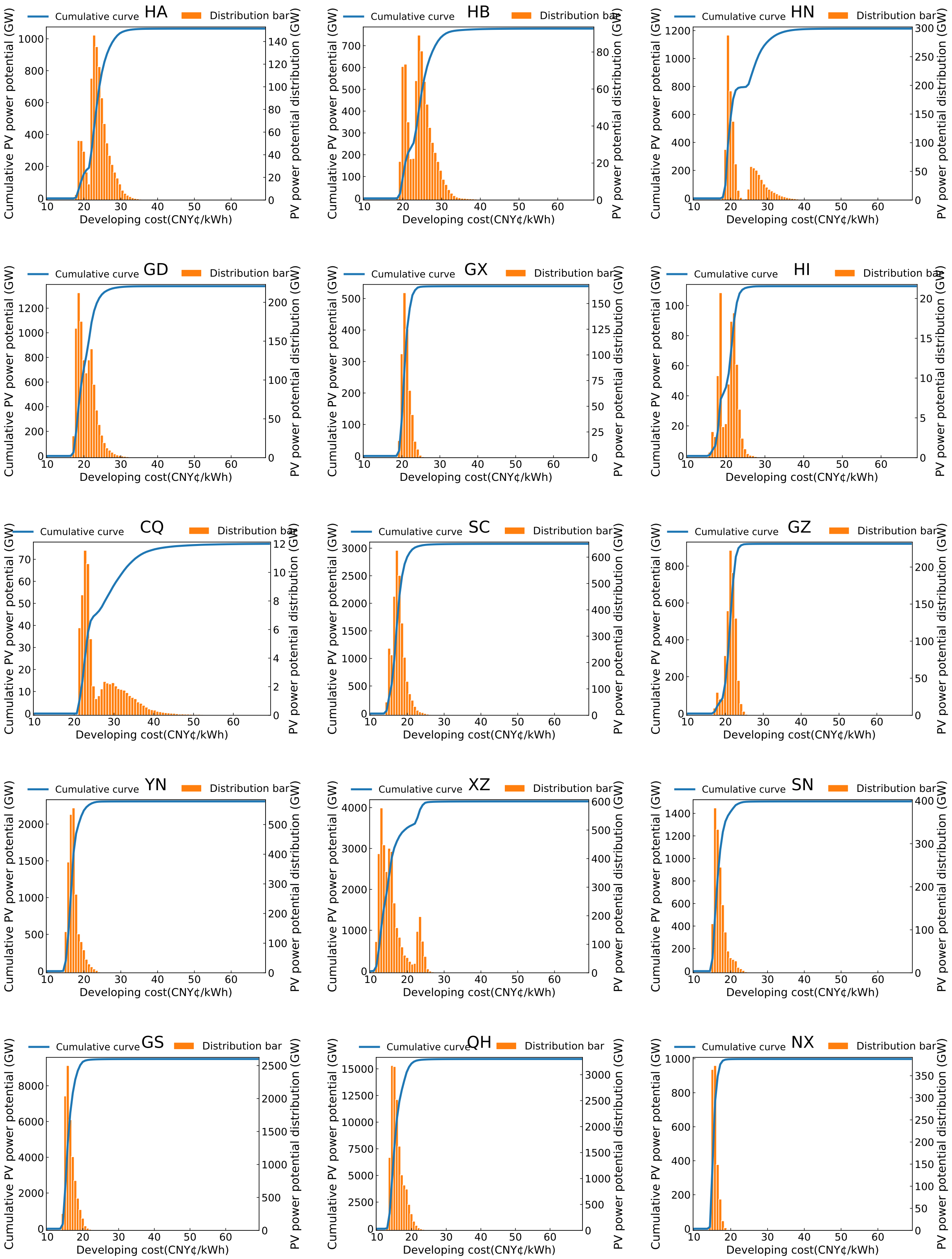
HA
HB
HN
GD
GX
HI
CQ
SC
GZ
YN
XZ
SN
GS
QH
NX
Cumulative curve
Distribution bar
Cumulative PV power potential (GW)
PV power potential distribution (GW)
Developing cost(CNY¢/kWh)

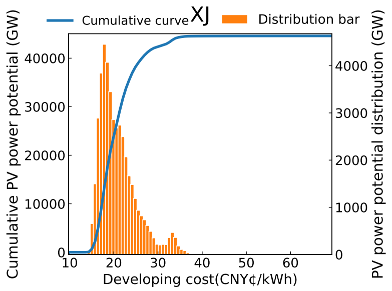

Supplementary Figure 8. **Supply curves of PV power in each province**

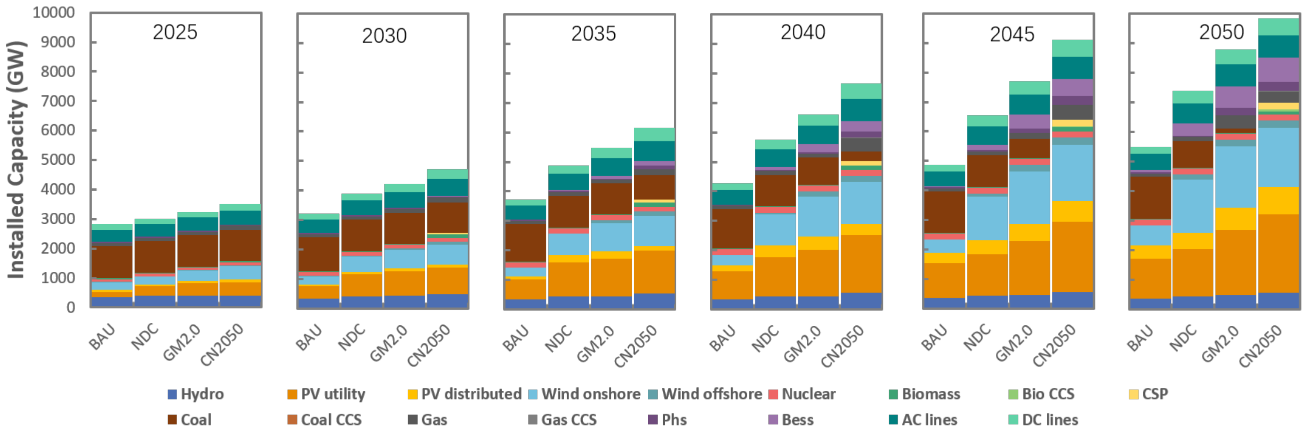

Supplementary Figure 9. Capacity mix in China under different carbon emission target scenarios

446 The six charts present the capacity mix in corresponding target years for each scenario. Both the installed capacity of AC
447 and DC inter-provincial transmission lines are also shown.

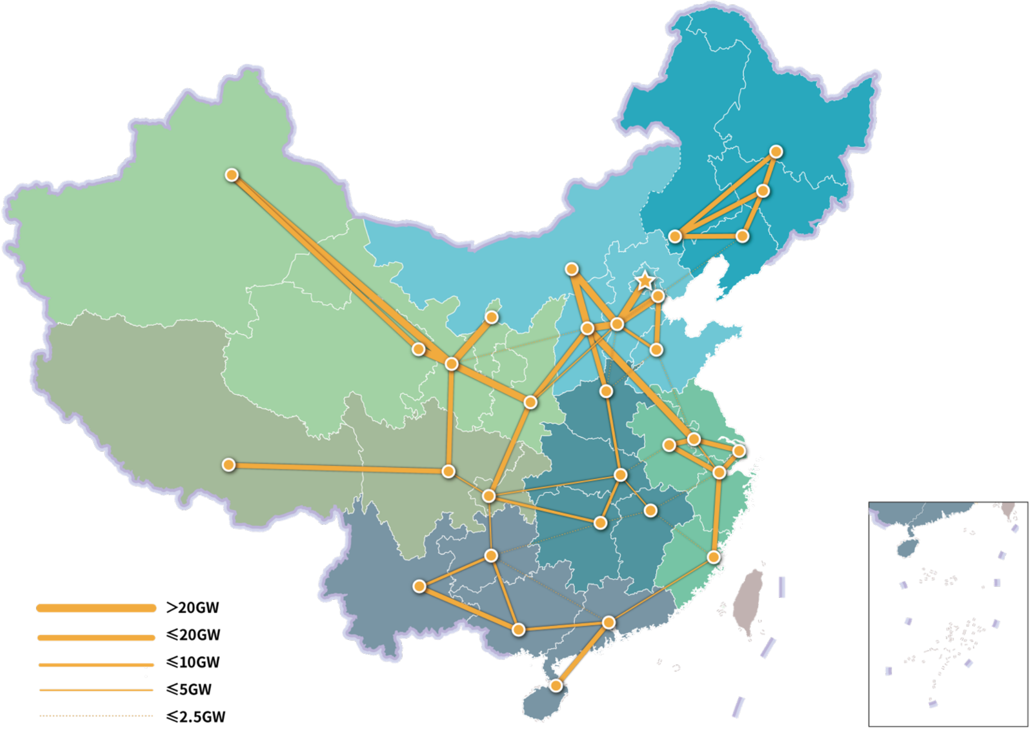

(a) China's inter-provincial AC transmission line capacity in 2050

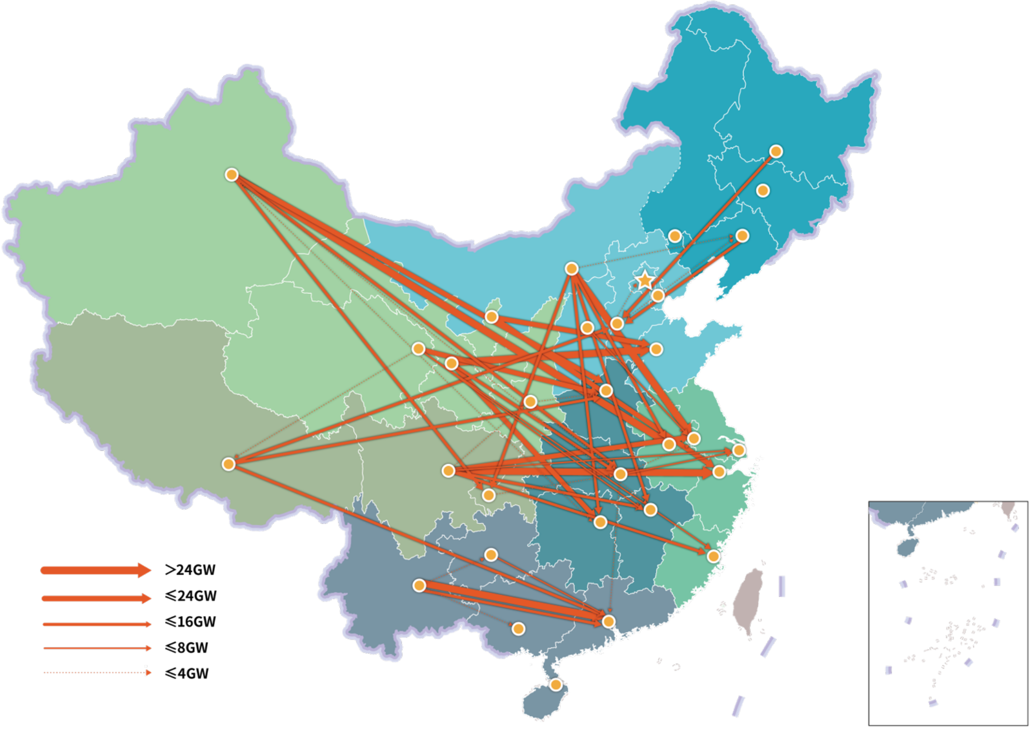

(b) China's inter-provincial DC transmission line capacity in 2050

Supplementary Figure 10. Transmission network topology under carbon neutrality goals in 2050

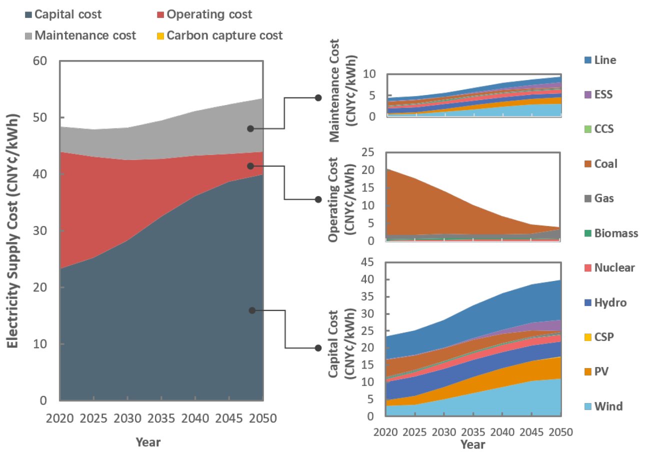

(a) GM2.0 scenarios

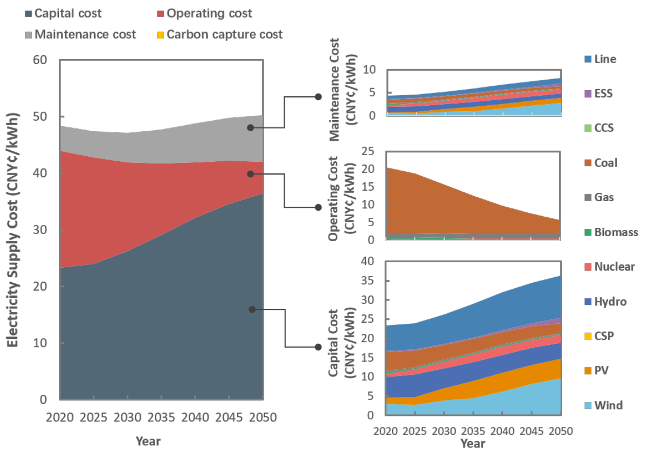

(b) NDC scenarios

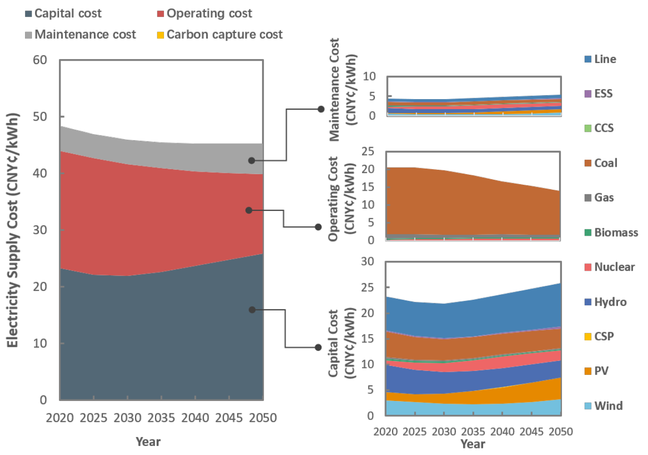

(c) BAU scenarios

Supplementary Figure 11. **Composition variation of electricity supply cost**

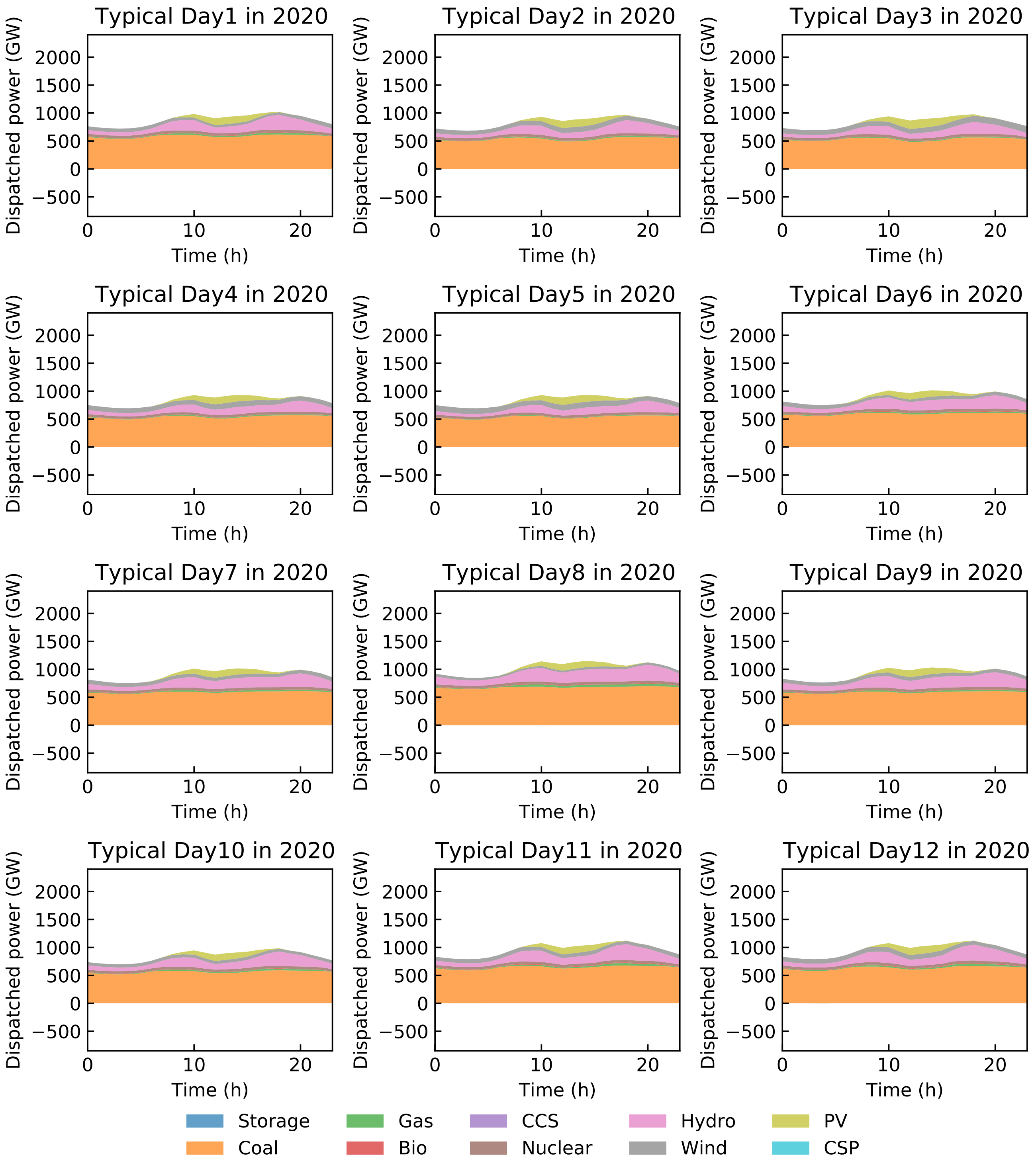

Supplementary Figure 12. Hourly dispatch schedules in 2020

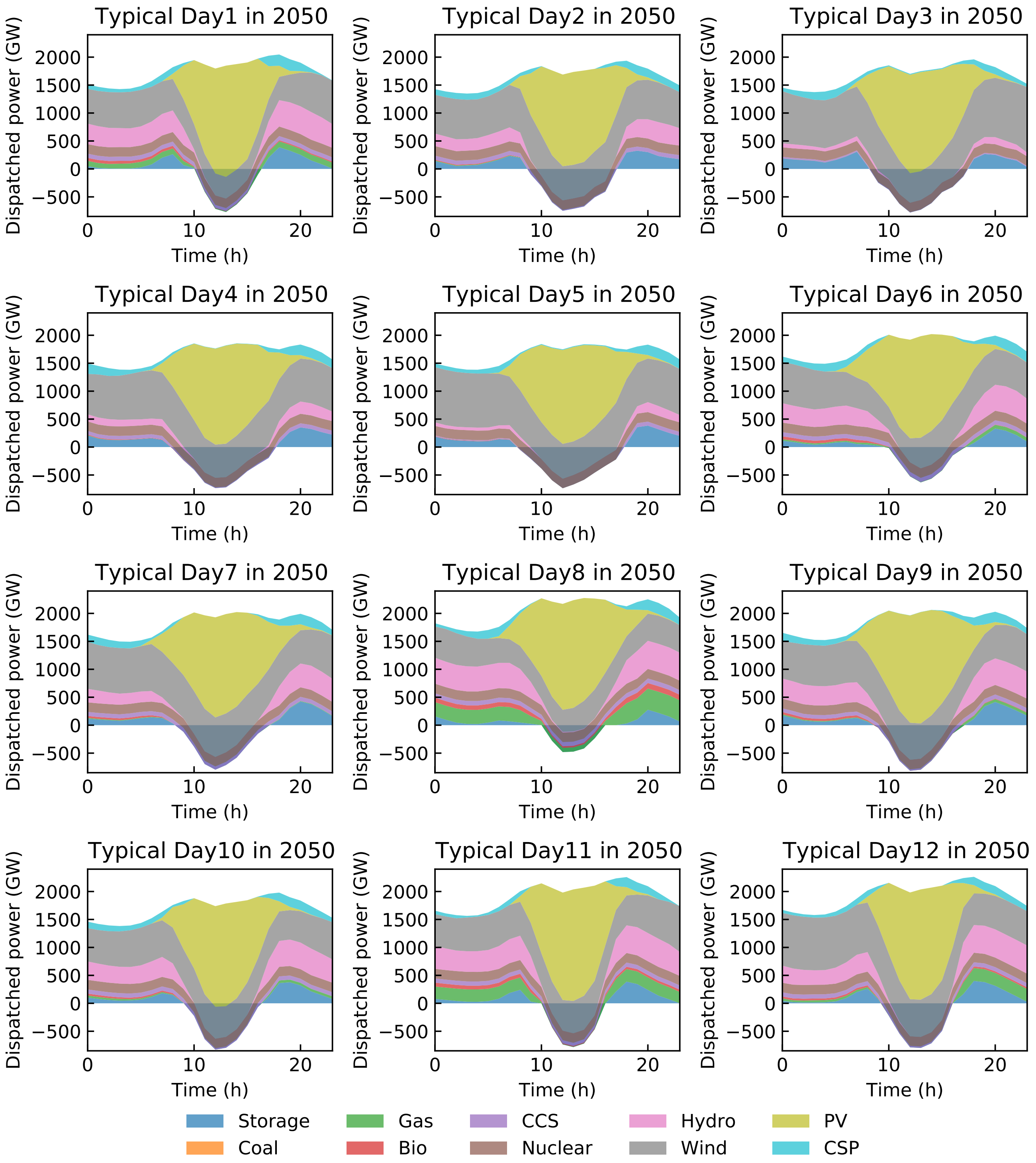

Supplementary Figure 13. **Hourly dispatch schedules in 2050 under CN2050 scenario**

Supplementary Tables

Supplementary Table 6. Abbreviations and regions of provinces in Mainland China

Province name	Abbreviation of province name	Region	Abbreviation of region
Beijing	BJ	North China	N
Tianjin	TJ	North China	N
Hebei	HE	North China	N
Shanxi	SX	North China	N
Inner Mongolia	NM	North China	N
Liaoning	LN	Northeast China	NE
Jilin	JL	Northeast China	NE
Heilongjiang	HL	Northeast China	NE
Shanghai	SH	East China	E
Jiangsu	JS	East China	E
Zhejiang	ZJ	East China	E
Anhui	AH	East China	E
Fujian	FJ	East China	E
Jiangxi	JX	Central China	C
Shandong	SD	North China	N
Henan	HA	Central China	C
Hubei	HB	Central China	C
Hunan	HN	Central China	C
Guangdong	GD	South China	S
Guangxi	GX	South China	S
Hainan	HI	South China	S
Chongqing	CQ	Southwest China	SW
Sichuan	SC	Southwest China	SW
Guizhou	GZ	South China	S
Yunnan	YN	South China	S
Tibet	XZ	Southwest China	SW
Shaanxi	SN	Northwest China	NW
Gansu	GS	Northwest China	NW
Qinghai	QH	Northwest China	NW
Ningxia	NX	Northwest China	NW
Xinjiang	XJ	Northwest China	NW

Supplementary Table 7. The R-squared value of the transmission line length fitting results for each region

Region	750kV lines	500kV lines	330kV lines	220kV lines
N	N/A	0.940876	N/A	0.973249
NE	N/A	0.975044	N/A	0.968192
E	N/A	0.947023	N/A	0.985126
C	N/A	0.956851	N/A	0.944549
S	N/A	0.978517	N/A	0.983638
SW	N/A	0.982999	N/A	0.982782
NW	0.970405	N/A	0.973176	0.962292

Supplementary Table 8. The R-squared value of the transformer capacities fitting results for each region

Region	750kV Transformer	500kV Transformer	330kV Transformer	220kV Transformer
N	N/A	0.951368	N/A	0.975953
NE	N/A	0.940876	N/A	0.948285
E	N/A	0.963468	N/A	0.98402
C	N/A	0.918828	N/A	0.938904
S	N/A	0.980162	N/A	0.9771
SW	N/A	0.991865	N/A	0.993964
NW	0.95517	N/A	0.969943	0.964588

The training set includes annual installed transformer capacity and transmission line length data at each voltage level (750 kV, 500 kV, 330 kV, and 220 kV) in each province from 2008 to 2018[32]. The historical (from 2008 to 2018) annual electricity load demands and local power generation levels are also collected and used to regress the corresponding relationships with installed transformer capacity and transmission line length. R-squared is a metric to present the fitting goodness of local grid development results. It is also known as the coefficient of determination. Numerically, it is the square of the sample Pearson correlation coefficient. An R-squared value of 1 indicates that the regression prediction results perfectly fit the original data. R-squared is calculated using the "ElasticNet" Function in the sklearn package. The data of the training set are provided in a general public data repository[33].

Supplementary Table 9. Projection results of within-province transformer capacities in CN2050 (GW)

Year	750kV Transformer	500kV Transformer	330kV Transformer	220kV Transformer
2025	234.25	2136.98	182.57	3304.01
2030	359.14	2900.18	269.31	4424.06
2035	590.40	3631.27	380.66	5554.29
2040	895.85	4554.13	472.95	6953.97
2045	1048.33	5458.98	543.07	8383.83
2050	1096.01	5777.24	602.45	8889.27

Supplementary Table 10. Projection results of within-province transformer lines in CN2050 (km)

Year	750kV lines	500kV lines	330kV lines	220kV lines
2025	31944.13	258950.48	37471.26	621677.42
2030	49520.97	344659.98	51737.87	828070.82
2035	71424.65	424814.34	73282.20	1035205.82
2040	89294.89	515610.87	95038.20	1255482.88
2045	104165.64	611945.05	107041.01	1495513.49
2050	117809.07	641369.87	113015.99	1576133.51

Supplementary Table 11. The data source of Renewable Energy Resources in GREAN platform

Data Description	Data Source, spatial resolution and data type
Global mesoscale wind resources data	Wind resource data is calculated and produced by Vortex, with a resolution of 9km×9km. (Raster data).
Global solar energy resource data	Solar resource data is calculated and produced by SolarGIS, with a resolution of 9km×9km. (Raster data)

Supplementary Table 12. The data source of geographic information Resources in GREAN platform

Data Description	Data Source, spatial resolution and data type
Classification information of global land covers	Land cover data covers the land range from 80° degrees north latitude to 80° south latitude released by National Geomatics Center of China, with a resolution of 30m×30m. (Raster data).
Global distribution of major reservoirs	Reservoirs data is from the global water system projects in Bonn, Germany, including more than 6,500 artificial reservoirs with a cumulative storage capacity of about 6.2 trillion m3 (Raster data).
Global distribution of lakes and wetlands	Lakes and wetlands data is jointly developed by the World Wide Fund for Nature, the Environmental Systems Research Center and Kassel University in Germany, including lakes and permanent open water bodies other than artificial reservoirs with a resolution of 1 km×1 km. (Raster data).
Global distribution of major geological faults	Geological faults data is from the American Environment Systems Research Institute. (Vector data).
Global distribution of plate boundaries	Plate boundary data is from the American Environmental Systems Research Institute. (Vector data)
Global distribution of historical seismic activity frequency	Historical earthquake frequency data is from the World Resources Institute (WRI), including the geographical distribution of earthquakes with magnitude 4.5 or higher since 1976 with a resolution of 5 km×5 km. (Raster data)
Global distribution of main stratum	Major stratum data is from the joint research results of European Commission, German Federal Ministry of Education and Research, German Science Foundation and other institutions. (Vector data)
Global terrain elevation data	Global terrain elevation data is from the digital products of National Aeronautics and Space Administration (NASA) and Ministry of Economy Trade and Industry (METI) with a resolution of 30m×30m. (Raster data)
Global ocean boundaries data	Ocean boundary is from the Flanders Marine Institute (VLIZ) in Belgium, including the 200-nautical-mile exclusive economic zone, 24-nautical-mile contiguous zone, 12-nautical-mile territorial sea area and other information stipulated in the United Nations Convention on the Law of the Sea. (Vector data)

Supplementary Table 13. The data source of human activities information in GREAN platform

Data Description	Data Source, spatial resolution and data type
Global distribution of major conservation areas	Conservation areas is from the data set jointly released by the International Union for Conservation of Nature (IUCN) and the World Conservation Monitoring Center of the United Nations Environment Programme (UNEP-WCMC). (Vector data)
Global population distribution	Population distribution data is from Columbia University's International Geoscience Information Network Center, including the population distribution data in 2000, 2005, 2010 and 2015, with a resolution of 900m×900m. (Raster data)
Global distribution of transportation infrastructure	Transportation infrastructure is from the global railway, airport and port data set released by the North American Cartographic Information Society (NACIS) and the global road network data set released by the Socioeconomic Data and Applications Center of NASA. (Vector data)
Geographic distribution of global power grid	Global grid geographic wiring diagram is from the Global Energy Interconnection Development and Cooperation Organization, covering the backbone transmission network data of 147 countries in Europe, Asia, America, Africa and Oceania as of 2017, including 110kV-1000kV AC power grids and major DC transmission projects. (Vector data)
Global power plant information and geographic distribution	Power plant information and geographical distribution data is from the joint research results of Google, Royal Institute of Technology in Stockholm and World Resources Institute (WRI), including the location distribution and installed capacity of global power plants of 2017. (Vector data)

Supplementary Table 14. **The parameter of generation technologies**

Type	Start-up cost (10^4CNY/ MW)	Maintenance cost (10^4CNY/ MW/a)	Variable cost (10^4CNY/ MWh)	Investment cost (CNY/ kW)	Carbon emission (t/MWh)	Maximum ramp rate (%/min)	Minimum output rate (%)	Lifetime (a)	Water consumption (t/MWh)	Inertia constant (s)
Hydro	0.00	25.93	0.0000	14561	0	100%	0%	50	0	2.83
PV utility	0.00	6.65	0.0000	4599	0	100%	0%	25	0	0
Onshore wind	0.00	14.60	0.0000	7600	0	100%	0%	25	0	0
Nuclear	2.20	55.64	0.0048	16000	0	0%	50%	50	4.167	4.07
Biomass	0.05	44.80	0.0506	10528	0.35	10%	35%	40	3.32	2.94
CSP	0.00	54.05	0.0000	27500	0	100%	10%	35	3.13	2.94
Coal	0.11	6.20	0.0274	4046	0.865	2%	40%	40	3.82	5.89
Coal CCS	0.11	24.40	0.0296	8009	0.865	2%	40%	40	5.02	5.89
Gas	0.03	9.80	0.0447	2387	0.312	7%	30%	40	0.97	4.97
Gas CCS	0.03	28.00	0.0499	6020	0.312	7%	30%	40	1.4	4.97
Bio CCS	0.05	44.80	0.0506	22319	0.35	10%	35%	40	4.007	2.94
PV distributed	0.00	6.30	0.0000	4599	0	100%	0%	25	0	0
Offshore wind	0.00	43.45	0.0000	17800	0	100%	0%	25	0	0

Supplementary Table 15. **The parameter of energy storage technologies**

Type	Maintenance cost (10^4CNY/ MW/a)	Capital cost of storage power (CNY/kW)	Capital cost of storage energy (CNY/kWh)	Efficiency (%)	Maximum power discharge time (h)	Lifetime (a)	Inertia constant (s)
Pump storage	14.92	5969	N/A	0.75	8	50	2.83
Battery	16	3200	1600	0.9	2	15	5.89

Supplementary Table 16. Variable costs of thermal units in different provinces (CNY/kWh)

Province Name	Coal	Gas	Biomass	Coal-CCS	Gas-CCS	Bio-CCS
Beijing	0.264	0.487	0.570	0.327	0.524	0.623
Tianjin	0.264	0.487	0.570	0.327	0.524	0.623
Hebei	0.244	0.482	0.570	0.307	0.519	0.623
Shanxi	0.245	0.464	0.570	0.308	0.501	0.623
Inner Mongolia	0.213	0.324	0.570	0.276	0.362	0.623
Liaoning	0.276	0.482	0.533	0.339	0.519	0.583
Jilin	0.273	0.431	0.533	0.336	0.468	0.583
Heilongjiang	0.278	0.431	0.420	0.341	0.468	0.459
Shanghai	0.302	0.533	0.474	0.365	0.570	0.518
Jiangsu	0.290	0.528	0.531	0.353	0.565	0.581
Zhejiang	0.306	0.530	0.531	0.369	0.568	0.581
Anhui	0.284	0.510	0.531	0.347	0.547	0.581
Fujian	0.288	0.533	0.531	0.351	0.570	0.581
Jiangxi	0.307	0.477	0.542	0.370	0.514	0.592
Shandong	0.302	0.482	0.535	0.365	0.519	0.585
Henan	0.267	0.490	0.562	0.330	0.527	0.615
Hubei	0.319	0.477	0.414	0.382	0.514	0.453
Hunan	0.333	0.477	0.542	0.396	0.514	0.592
Guangdong	0.322	0.533	0.544	0.385	0.570	0.595
Guangxi	0.295	0.490	0.544	0.358	0.527	0.595
Hainan	0.320	0.401	0.544	0.383	0.438	0.595
Chongqing	0.303	0.401	0.387	0.367	0.438	0.423
Sichuan	0.321	0.403	0.513	0.384	0.440	0.561
Guizhou	0.252	0.419	0.568	0.315	0.456	0.621
Yunnan	0.303	0.419	0.448	0.366	0.456	0.489
Tibet	0.248	0.533	0.513	0.311	0.570	0.561
Shaanxi	0.248	0.324	0.508	0.311	0.362	0.556
Gansu	0.228	0.347	0.508	0.291	0.384	0.556
Qinghai	0.218	0.368	0.508	0.281	0.405	0.556
Ningxia	0.198	0.307	0.508	0.261	0.344	0.556
Xinjiang	0.176	0.276	0.508	0.239	0.313	0.556

The data of the Supplementary Table 16 refer to Supplementary Refs. 14, 34, 35.

Supplementary Table 17. The parameter of existing AC transmission lines in 2020

Num	From Bus	To bus	Reactance (p.u.)	Investment cost (10^4 CNY/MW)	length (km)	Capacity (MW)	Voltage (kV)	Lifetime (a)	loss rate
1	SX	HA	8.93E-03	84.30	362.00	5000	1000	40	1.50%
2	HA	HB	2.51E-03	59.79	226.23	9800	1000	40	1.16%
3	AH	ZJ	1.42E-03	78.24	370.81	14800	1000	40	1.62%
4	ZJ	SH	1.00E-03	50.84	121.67	13600	1000	40	1.69%
5	AH	JS	1.66E-03	73.03	340.04	14600	1000	40	1.71%
6	JS	SH	1.56E-03	67.28	285.12	14600	1000	40	1.71%
7	ZJ	FJ	1.83E-03	88.58	468.88	14100	1000	40	1.70%
8	NM	HE	1.19E-03	76.23	371.51	29960	1000	40	1.89%
9	HE	TJ	8.74E-04	60.33	180.37	24800	1000	40	1.87%
10	TJ	SD	3.43E-03	74.63	278.00	10000	1000	40	2.00%
11	NM	SX	1.42E-03	70.94	246.00	20000	1000	40	2.00%
12	SX	HE	9.39E-04	65.14	243.66	30800	1000	40	1.77%
13	SN	SX	3.39E-03	74.28	275.00	10000	1000	40	2.00%
14	HE	SD	2.24E-03	106.77	583.52	24800	1000	40	1.87%
15	HE	BJ	1.11E-03	68.24	258.82	27200	1000	40	1.83%
16	GZ	GX	1.50E-02	168.99	966.67	7200	500	40	2.00%
17	GX	GD	3.67E-03	102.86	544.50	9600	500	40	2.35%
18	YN	GX	5.71E-03	58.99	264.50	4800	500	40	2.35%
19	GD	HI	1.10E-02	90.62	233.20	1200	500	40	2.00%
20	HE	HA	6.48E-03	30.52	69.00	1000	500	40	2.00%
21	SX	JS	2.39E-02	113.05	508.00	2000	500	40	3.00%
22	SN	HE	1.03E-02	100.08	439.00	4000	500	40	2.50%
23	GZ	HN	6.35E-04	65.77	70.00	2500	220	40	2.00%
24	SC	XZ	9.48E-02	333.70	1009.00	600	500	40	2.00%
25	SN	GS	3.22E-03	78.57	310.00	10000	750	40	0.83%
26	GS	NX	1.14E-03	62.72	167.45	10000	750	40	0.83%
27	GS	QH	2.19E-03	77.75	302.64	15000	750	40	0.83%
28	GS	XJ	6.00E-03	103.18	531.30	10000	750	40	0.83%
29	NM	HL	1.06E-02	52.94	225.90	2400	500	40	1.92%
30	NM	JL	7.08E-03	52.94	225.90	3600	500	40	1.92%
31	NM	LN	3.54E-03	52.94	225.90	7200	500	40	1.92%
32	HL	JL	5.31E-03	52.94	225.90	4800	500	40	1.92%
33	JL	LN	5.31E-03	52.94	225.90	4800	500	40	1.92%
34	JS	ZJ	2.43E-03	29.72	77.70	3600	500	40	0.83%
35	HB	HN	5.23E-03	43.73	167.10	3600	500	40	0.80%
36	HB	JX	5.23E-03	43.73	167.10	3600	500	40	0.80%
37	HB	CQ	3.93E-03	43.73	167.10	4800	500	40	0.80%
38	SC	CQ	3.93E-03	43.73	167.10	4800	500	40	0.80%
39	BJ	TJ	3.76E-03	30.08	80.00	2400	500	40	1.34%
40	LN	HE	2.63E-03	28.08	56.00	2000	500	40	1.00%
41	GZ	CQ	4.98E-03	32.65	106.00	2640	500	40	2.00%

Supplementary Table 18. **The parameter of candidate AC transmission lines**

Num	From Bus	To bus	Reactance (p.u.)	Investment cost (10^4 CNY/MW)	length (km)	Capacity (MW)	Voltage (kV)	Lifetime (a)	loss rate
1	HB	HA	6.48E-04	91.75	336.32	59600	1000	40	1.66%
2	HB	JX	8.89E-04	80.03	280.80	29600	1000	40	1.82%
3	JX	HN	2.14E-03	96.81	347.30	20000	1000	40	1.50%
4	SD	HA	2.15E-03	96.94	348.40	20000	1000	40	1.50%
5	SN	SX	1.17E-03	108.22	446.35	40000	1000	40	1.50%
6	SX	JS	4.68E-03	144.16	758.30	20000	1000	40	1.50%
7	FJ	GD	5.91E-03	62.82	251.60	4800	500	40	2.50%
8	SC	CQ	5.40E-04	63.14	133.51	29600	1000	40	1.82%
9	SN	CQ	1.75E-03	89.51	283.93	20000	1000	40	1.50%
10	GS	SC	1.84E-03	91.18	298.40	20000	1000	40	1.50%
11	GS	SN	1.58E-03	86.36	256.63	20000	1000	40	1.50%
12	XJ	QH	4.07E-03	138.63	717.85	20000	750	40	2.00%
13	XJ	GS	4.57E-03	148.48	806.44	20000	750	40	2.00%
14	QH	GS	5.33E-04	69.24	93.91	20000	750	40	2.00%
15	GS	NX	9.99E-04	78.39	176.16	20000	750	40	2.00%
16	XZ	SC	7.68E-03	114.30	622.38	10000	1000	40	1.50%
17	CQ	HB	4.43E-03	82.52	377.39	9600	500	40	2.50%
18	HB	HN	1.68E-03	45.84	143.26	9600	500	40	2.50%
19	YN	GZ	2.54E-03	57.33	216.60	9600	500	40	2.50%
20	YN	GX	3.64E-03	71.92	309.69	9600	500	40	2.50%
21	GD	HI	2.69E-03	59.34	229.42	9600	500	40	2.50%
22	FJ	ZJ	1.59E-03	103.35	488.00	30600	1000	40	1.81%
23	AH	ZJ	1.20E-03	94.17	399.15	29600	1000	40	1.82%
24	AH	JS	6.39E-04	81.13	318.37	31600	1000	40	1.80%
25	NM	HE	1.35E-03	96.53	415.93	29600	1000	40	1.82%
26	HE	BJ	5.39E-04	79.67	245.18	49600	1000	40	1.69%
27	HE	TJ	3.93E-04	70.16	162.86	49600	1000	40	1.69%
28	TJ	BJ	6.36E-04	31.89	54.19	9600	500	40	2.50%
29	SX	HE	3.07E-04	70.14	199.84	59200	1000	40	1.82%
30	HE	SD	8.77E-04	116.67	566.42	49600	1000	40	1.69%
31	JL	LN	1.65E-03	45.46	140.78	9600	500	40	2.50%
32	NM	LN	5.78E-03	100.56	492.50	9600	500	40	2.50%
33	HL	JL	1.36E-03	41.51	115.57	9600	500	40	2.50%
34	NM	HL	7.80E-03	127.42	663.99	9600	500	40	2.50%
35	NM	JL	6.90E-03	115.40	587.26	9600	500	40	2.50%
36	SX	HA	4.47E-03	98.50	362.00	10000	1000	40	1.50%
37	ZJ	SH	8.48E-04	72.64	137.50	20000	1000	40	1.50%
38	JS	SH	2.18E-03	93.77	353.00	22000	1000	40	1.50%
39	TJ	SD	1.71E-03	88.83	278.00	20000	1000	40	1.50%
40	NM	SX	7.10E-04	85.14	246.00	40000	1000	40	1.50%

Supplementary Table 19. The parameter of existing DC transmission lines in 2020

Num	From Bus	To bus	Capital cost (10^4 CNY/MW)	length (km)	Capacity (MW)	Voltage (kV)	Lifetime (a)	loss rate
1	HB	JS	126.91	370	3000	±500	40	7.50%
2	HB	SH	189.45	202	7223	±500	40	7.50%
3	HB	GD	185.36	1238	3000	±500	40	7.65%
4	NM	LN	163.14	908	3000	±500	40	4.12%
5	QH	XZ	451.46	1038	600	±400	40	13.70%
6	SC	JS	227.49	2100	7200	±800	40	7.00%
7	SC	SH	230.07	1907	6400	±800	40	7.00%
8	SC	ZJ	199.79	1679.6	7500	±800	40	6.50%
9	SN	HA	42.60	0	390	±330	40	1.00%
10	SC	SN	137.96	534	3000	±500	40	3.00%
11	HE	LN	42.60	0	300	±125	40	1.70%
12	XJ	HA	220.27	2192	8000	±800	40	7.20%
13	NX	SD	244.50	1333	4000	±660	40	7.00%
14	NX	ZJ	196.14	1720	8000	±800	40	6.50%
15	GS	HN	230.03	2383	8000	±800	40	6.50%
16	YN	GD	211.29	347	21400	±1000	40	6.55%
17	NM	SD	158.59	409	20000	±800	40	6.75%
18	NM	JS	174.79	1628	10000	±800	40	7.00%
19	SX	JS	165.41	1119	8000	±800	40	7.00%
20	XJ	AH	314.12	3324	12000	±1100	40	7.00%
21	CQ	HB	102.00	0	2600	±500	40	0.70%
22	GZ	GD	153.17	202	7800	±500	40	6.72%
23	YN	GX	171.75	1105	3200	±500	40	6.50%
24	QH	HA	189.34	1587	8000	±800	40	6.50%
25	YN	GZ	42.60	0	3000	±350	40	1.00%
26	HE	BJ	119.64	262	3000	±500	40	6.00%

Supplementary Table 20. The parameter of candidate DC transmission lines

Num	From Bus	To bus	Capital cost (10^4 CNY/MW)	length (km)	Capacity (MW)	Voltage (kV)	Lifetime (a)	loss rate
1	YN	GD	182.43	1452.00	16000	±800	40	6.50%
2	XJ	SC	263.47	2456.00	24000	±1100	40	6.50%
3	SC	ZJ	203.76	409.00	32000	±800	40	6.50%
4	SC	JS	219.24	2172.00	16000	±800	40	6.50%
5	SN	HB	166.33	1137.00	16000	±800	40	6.50%
6	SC	JX	195.67	1711.00	16000	±800	40	6.50%
7	XJ	HA	232.77	2436.54	16000	±800	40	6.50%
8	XJ	AH	256.24	2895.58	16000	±800	40	6.50%
9	XJ	CQ	220.68	2200.00	16000	±800	40	6.50%
10	XJ	HB	249.49	2763.52	16000	±800	40	6.50%
11	XJ	JX	262.22	3012.61	16000	±800	40	6.50%
12	XJ	HN	253.44	2840.90	16000	±800	40	6.50%
13	NX	SD	157.62	966.60	16000	±800	40	6.50%
14	NX	ZJ	188.33	1567.34	16000	±800	40	6.50%
15	NX	FJ	202.25	1839.56	16000	±800	40	6.50%
16	GS	HN	171.13	1230.93	16000	±800	40	6.50%
17	GS	HB	167.39	1157.69	16000	±800	40	6.50%
18	QH	HA	164.12	1093.70	16000	±800	40	6.50%
19	QH	FJ	211.95	2029.29	16000	±800	40	6.50%
20	QH	JX	189.28	1585.94	16000	±800	40	6.50%
21	YN	GD	163.71	1085.77	16000	±800	40	6.50%
22	GZ	GD	147.01	759.05	16000	±800	40	6.50%
23	SC	FJ	188.69	1574.30	16000	±800	40	6.50%
24	NM	CQ	176.28	1331.57	16000	±800	40	6.50%
25	NM	JS	167.77	1165.23	16000	±800	40	6.50%
26	NM	AH	165.24	1115.61	16000	±800	40	6.50%
27	NM	JX	179.97	1403.89	16000	±800	40	6.50%
28	NM	HN	180.16	1407.56	16000	±800	40	6.50%
29	GS	SD	150.43	826.00	16000	±800	40	6.50%
30	HL	HE	175.67	1319.65	16000	±800	40	6.50%
31	LN	HE	152.50	866.47	16000	±800	40	6.50%
32	XZ	HA	200.23	1800.00	16000	±800	40	6.50%
33	XZ	HE	209.42	1979.80	16000	±800	40	6.50%
34	XZ	GD	203.42	1862.40	16000	±800	40	6.50%

Supplementary Table 21. Annual load demands at the provincial level in the NDC/BAU scenario (TWh)

Province Name	2020	2025	2030	2035	2040	2045	2050
Beijing	119.07	128.29	145.23	156.90	164.72	172.25	179.47
Tianjin	93.88	101.15	114.51	123.70	129.87	135.81	141.50
Hebei	386.98	416.92	472.00	509.90	535.33	559.81	583.27
Shanxi	227.95	245.58	278.03	300.35	315.33	329.75	343.57
Inner Mongolia	365.77	394.08	446.14	481.96	506.00	529.14	551.31
Liaoning	241.20	251.22	278.88	295.42	305.58	314.83	323.19
Jilin	76.87	80.06	88.87	94.14	97.38	100.33	102.99
Heilongjiang	100.72	104.90	116.46	123.36	127.60	131.47	134.96
Shanghai	159.67	179.61	200.37	213.29	220.63	227.31	233.35
Jiangsu	688.10	774.04	863.50	919.21	950.83	979.63	1005.63
Zhejiang	514.38	578.62	645.50	687.15	710.78	732.31	751.75
Anhui	249.43	280.58	313.01	333.20	344.66	355.11	364.53
Fujian	246.26	277.02	309.04	328.98	340.29	350.60	359.91
Jiangxi	157.88	175.07	199.17	216.22	229.26	242.12	254.77
Shandong	655.27	705.97	799.24	863.41	906.48	947.92	987.64
Henan	333.97	370.33	421.31	457.37	484.95	512.16	538.92
Hubei	227.32	252.07	286.77	311.32	330.09	348.61	366.83
Hunan	190.55	211.29	240.38	260.96	276.69	292.22	307.49
Guangdong	702.67	790.43	921.46	1025.05	1086.86	1147.84	1207.81
Guangxi	199.81	224.76	262.02	291.48	309.06	326.40	343.45
Hainan	37.52	42.21	49.21	54.74	58.04	61.30	64.50
Chongqing	121.64	140.13	164.96	185.29	200.38	215.83	231.63
Sichuan	275.66	317.56	373.82	419.91	454.09	489.11	524.91
Guizhou	153.73	172.93	201.60	224.26	237.79	251.13	264.25
Yunnan	180.24	202.75	236.36	262.93	278.78	294.42	309.81
Tibet	10.60	12.21	14.38	16.15	17.46	18.81	20.19
Shaanxi	194.85	223.40	269.43	310.07	350.42	394.44	442.38
Gansu	129.88	148.91	179.59	206.68	233.57	262.92	294.87
Qinghai	74.22	85.09	102.62	118.10	133.47	150.24	168.50
Ningxia	108.67	124.60	150.27	172.94	195.44	219.99	246.73
Xinjiang	286.26	328.21	395.84	455.55	514.82	579.49	649.92

Supplementary Table 22. **Annual load demands at the provincial level in the GM2.0 scenario (TWh)**

Province Name	2020	2025	2030	2035	2040	2045	2050
Beijing	119.07	130.67	147.06	160.21	170.88	181.21	191.14
Tianjin	93.88	103.03	115.95	126.32	134.73	142.87	150.71
Hebei	386.98	424.67	477.94	520.67	555.36	588.90	621.20
Shanxi	227.95	250.15	281.53	306.69	327.13	346.89	365.91
Inner Mongolia	365.77	401.40	451.75	492.14	524.93	556.64	587.17
Liaoning	241.20	255.89	282.39	301.65	317.01	331.20	344.21
Jilin	76.87	81.55	89.99	96.13	101.03	105.55	109.69
Heilongjiang	100.72	106.85	117.92	125.97	132.38	138.30	143.74
Shanghai	159.67	182.95	202.89	217.80	228.89	239.13	248.52
Jiangsu	688.10	788.42	874.37	938.62	986.40	1030.55	1071.04
Zhejiang	514.38	589.38	653.62	701.65	737.37	770.37	800.64
Anhui	249.43	285.80	316.95	340.24	357.56	373.56	388.24
Fujian	246.26	282.17	312.93	335.92	353.03	368.82	383.32
Jiangxi	157.88	178.33	201.68	220.79	237.84	254.71	271.34
Shandong	655.27	719.09	809.29	881.64	940.39	997.18	1051.88
Henan	333.97	377.21	426.61	467.03	503.10	538.78	573.98
Hubei	227.32	256.76	290.38	317.89	342.44	366.73	390.69
Hunan	190.55	215.22	243.41	266.47	287.05	307.40	327.49
Guangdong	702.67	805.12	933.06	1046.69	1127.53	1207.49	1286.37
Guangxi	199.81	228.94	265.32	297.63	320.62	343.36	365.79
Hainan	37.52	42.99	49.83	55.89	60.21	64.48	68.69
Chongqing	121.64	142.73	167.03	189.21	207.87	227.05	246.69
Sichuan	275.66	323.46	378.52	428.77	471.08	514.53	559.05
Guizhou	153.73	176.15	204.14	229.00	246.68	264.18	281.43
Yunnan	180.24	206.52	239.33	268.48	289.21	309.73	329.96
Tibet	10.60	12.44	14.56	16.49	18.12	19.79	21.50
Shaanxi	194.85	227.55	272.82	316.62	363.53	414.94	471.15
Gansu	129.88	151.68	181.85	211.05	242.31	276.58	314.05
Qinghai	74.22	86.67	103.92	120.60	138.46	158.05	179.46
Ningxia	108.67	126.91	152.16	176.59	202.75	231.43	262.77
Xinjiang	286.26	334.31	400.82	465.16	534.08	609.61	692.19

Supplementary Table 23. **Annual load demands at the provincial level in the CN2050 scenario (TWh)**

Province Name	2020	2025	2030	2035	2040	2045	2050
Beijing	119.07	142.90	167.61	184.30	195.54	206.39	216.82
Tianjin	93.88	112.67	132.15	145.31	154.17	162.73	170.95
Hebei	386.98	464.41	544.73	598.96	635.49	670.75	704.66
Shanxi	227.95	273.56	320.87	352.81	374.33	395.10	415.08
Inner Mongolia	365.77	438.96	514.88	566.14	600.67	634.00	666.05
Liaoning	241.20	279.83	321.85	347.01	362.74	377.23	390.45
Jilin	76.87	89.18	102.57	110.59	115.60	120.21	124.43
Heilongjiang	100.72	116.85	134.40	144.91	151.48	157.52	163.05
Shanghai	159.67	200.07	231.24	250.55	261.91	272.36	281.91
Jiangsu	688.10	862.21	996.56	1079.76	1128.71	1173.77	1214.93
Zhejiang	514.38	644.53	744.97	807.17	843.76	877.44	908.21
Anhui	249.43	312.54	361.24	391.40	409.15	425.48	440.40
Fujian	246.26	308.58	356.66	386.44	403.96	420.08	434.81
Jiangxi	157.88	195.01	229.86	253.99	272.15	290.11	307.80
Shandong	655.27	786.38	922.39	1014.22	1076.06	1135.77	1193.20
Henan	333.97	412.51	486.23	537.26	575.68	613.66	651.09
Hubei	227.32	280.78	330.96	365.70	391.85	417.70	443.18
Hunan	190.55	235.36	277.42	306.54	328.46	350.13	371.48
Guangdong	702.67	880.47	1063.45	1204.08	1290.20	1375.31	1459.20
Guangxi	199.81	250.37	302.40	342.39	366.88	391.08	414.93
Hainan	37.52	47.02	56.79	64.30	68.90	73.44	77.92
Chongqing	121.64	156.09	190.37	217.66	237.86	258.60	279.83
Sichuan	275.66	353.73	431.42	493.25	539.04	586.04	634.16
Guizhou	153.73	192.63	232.66	263.43	282.27	300.89	319.25
Yunnan	180.24	225.84	272.78	308.85	330.94	352.77	374.29
Tibet	10.60	13.61	16.59	18.97	20.73	22.54	24.39
Shaanxi	194.85	248.85	310.95	364.23	415.98	472.61	534.45
Gansu	129.88	165.87	207.27	242.78	277.27	315.02	356.24
Qinghai	74.22	94.78	118.44	138.73	158.44	180.01	203.57
Ningxia	108.67	138.79	173.43	203.14	232.00	263.59	298.08
Xinjiang	286.26	365.60	456.83	535.11	611.13	694.33	785.18

Supplementary References

1. Heuberger, C. F., Staffell, I., Shah, N. & Mac Dowell, N. Impact of myopic decision-making and disruptive events in power systems planning. *Nat. Energy* **3**, 634–640 (2018).
2. Conejo, A. J., Cheng, Y., Zhang, N. & Kang, C. Long-term coordination of transmission and storage to integrate wind power. *CSEE J. Power Energy Syst.* **3**, 36–43 (2017).
3. He, G. *et al.* Rapid cost decrease of renewables and storage accelerates the decarbonization of china's power system. *Nat. Commun.* **11**, 2486–2494, DOI: 10.1038/s41467-020-16184-x (2020).
4. Li, Y. & McCalley, J. D. Design of a high capacity inter-regional transmission overlay for the us. *IEEE Transactions on Power Syst.* **30**, 513–521 (2014).
5. Romero, R., Monticelli, A., Garcia, A. & Haffner, S. Test systems and mathematical models for transmission network expansion planning. *IEE Proceedings-Generation, Transm. Distribution* **149**, 27–36 (2002).
6. National Development and Reform Commission. Typical power load curves in each provincial power system. in Chinese, https://www.ndrc.gov.cn/xxgk/zcfb/tz/201912/P020191230336066090861.pdf (2019).
7. China Electricity Council. *Annual development report of China's power industry 2021* (in Chinese, China Architecture & Building Press, 2021).
8. Irlam, L. Global costs of carbon capture and storage. https://www.globalccsinstitute.com/archive/hub/publications/201688/global-ccs-cost-updatev4.pdf (2017).
9. Faulstich, M. *et al.* Pathways towards a 100% renewable electricity system. https://www.umweltrat.de/SharedDocs/Downloads/EN/02_Special_Reports/2011_10_Special_Report_Pathways_renewables.pdf?__blob=publicationFile (2011).
10. Child, M., Kemfert, C., Bogdanov, D. & Breyer, C. Flexible electricity generation, grid exchange and storage for the transition to a 100% renewable energy system in europe. *Renew. energy* **139**, 80–101 (2019).
11. He, G. *et al.* Switch-china: a systems approach to decarbonizing china's power system. *Environ. science & technology* **50**, 5467–5473 (2016).
12. Chen, S., Liu, P. & Li, Z. Low carbon transition pathway of power sector with high penetration of renewable energy. *Renew. Sustain. Energy Rev.* **130**, 109985, DOI: 10.1016/j.rser.2020.109985 (2020).
13. Zhang, Q. & Chen, W. Modeling china's interprovincial electricity transmission under low carbon transition. *Appl. Energy* **279**, 115571, DOI: 10.1016/j.apenergy.2020.115571 (2020).
14. Ji, Z. *et al.* Low-carbon power system dispatch incorporating carbon capture power plants. *IEEE Transactions on Power Syst.* **28**, 4615–4623, DOI: 10.1109/TPWRS.2013.2274176 (2013).
15. China Power Engineering Consulting Group Co., Ltd. *Power Engineering Design Manual: Power System Planning and Design* (China Electric Power Press, Beijing, 2019).
16. Office of Gas and Electricity Markets. 9 august 2019 power outage report. https://www.ofgem.gov.uk/system/files/docs/2020/01/9_august_2019_power_outage_report.pdf (2019).
17. Fernandez-Guillamon, A., Gomez-Lazaro, E., Muljadi, E. & Molina-Garcia, A. Power systems with high renewable energy sources: A review of inertia and frequency control strategies over time. *Renew. Sustain. Energy Rev.* **115**, 109369, DOI: 10.1016/j.rser.2019.109369 (2019).
18. Boyd, S., Boyd, S. P. & Vandenberghe, L. *Convex optimization* (Cambridge university press, 2004).
19. Lofberg, J. Yalmip: A toolbox for modeling and optimization in matlab. In *2004 IEEE international conference on robotics and automation (IEEE Cat. No. 04CH37508)*, 284–289 (IEEE, 2004).
20. Heckman, J. Shadow prices, market wages, and labor supply. *Econom. journal econometric society* **42**, 679–694 (1974).
21. Conn, A., Gould, N. & Toint, P. A globally convergent lagrangian barrier algorithm for optimization with general inequality constraints and simple bounds. *Math. Comput.* **66**, 261–288 (1997).
22. National Renewable Energy Laboratory. 2021 annual technology baseline electricity data. https://atb.nrel.gov/electricity/2021/data (2021).
23. Xinhua News Agency. Large-scale wind and pv base projects in deserts, gobi and desert areas in my country started in an orderly manner. in Chinese, https://news.un.org/en/story/2020/09/1073052 (2021).
24. National Energy Administration. Circular on 2021 risk and early warning for coal power planning and construction. in Chinese, http://zfxxgk.nea.gov.cn/auto84/201805/t20180524_3186.htm (2018).

25. Sharifzadeh, M., Hien, R. K. T. & Shah, N. China's roadmap to low-carbon electricity and water: Disentangling greenhouse gas (ghg) emissions from electricity-water nexus via renewable wind and solar power generation, and carbon capture and storage. *Appl. Energy* **235**, 31–42, DOI: 10.1016/j.apenergy.2018.10.087 (2019).

26. Chen, X. *et al.* Power system capacity expansion under higher penetration of renewables considering flexibility constraints and low carbon policies. *IEEE Transactions on Power Syst.* **33**, 6240–6253 (2018).

27. Chen, X. *et al.* Pathway toward carbon-neutral electrical systems in china by mid-century with negative co2 abatement costs informed by high-resolution modeling. *Joule* **5**, 2715–2741, DOI: https://doi.org/10.1016/j.joule.2021.10.006 (2021).

28. National Renewable Energy Laboratory. 2020 annual technology baseline electricity data. https://www.nrel.gov/news/program/2020/2020-annual-technology-baseline-electricity-data-now-available.html (2020).

29. Wiser, R. *et al.* Expert elicitation survey predicts 37% to 49% declines in wind energy costs by 2050. *Nat. Energy* **6**, 555–565 (2021).

30. Graham, Paul and Hayward, Jenny and Foster, James and Havas, Lisa. Gencost project data. https://doi.org/10.25919/rpwh-wc51 (2021).

31. Global Energy Interconnection Development and Cooperation Organization. *Research on Global Renewable Energy Development and Investment (2020)* (China Electric Power Press, Beijing, 2020).

32. China Electricity Council. *China Electric Power Yearbook* (in Chinese, China Electric Power Press, Beijing, 2018).

33. Zhuo, Z. Source data for "cost increase in the electricity supply to achieve carbon neutrality in china", Figshare, DOI: 10.6084/m9.figshare.16929340 (2021).

34. Zhang, D. & Paltsev, S. The future of natural gas in china: Effects of pricing reform and climate policy. *Clim. Chang. Econ.* **7**, 1650012 (2016).

35. National Energy Administration. 2018 national electricity price regulatory bulletin. in Chinese, http://www.nea.gov.cn/138530255_15729388881531n.pdf (2019).
